# Chemometric Classification of *Mangifera indica* L. Leaf Cultivars, Based on Selected Phytochemical Parameters; Implications for Standardization of the Pharmaceutical Raw Materials

**DOI:** 10.1155/2023/7245876

**Published:** 2023-08-04

**Authors:** Bruhan Kaggwa, Maria Gloria Nakayita, Edson Ireeta Munanura, Henry Kyeyune, Clement Olusoji Ajayi, Raphael Wangalwa, Daniel Pillah Walakira, Godwin Anywar, Lynn K. Bagoloire, Teddy Kakazi, Gabriel Ddamulira, Fadhiru Pakoyo Kamba, Patrick Engeu Ogwang

**Affiliations:** ^1^Mbarara University of Science and Technology, Pharm-Bio Technology and Traditional Medicine Centre (PHARMBIOTRAC), P.O. Box 1410, Mbarara, Uganda; ^2^Makerere University, College of Health Sciences, Department of Pharmacy, P.O. Box 7062, Kampala, Uganda; ^3^Mbarara University of Science and Technology, Faculty of Science, Department of Biology, P.O. Box 1410, Mbarara, Uganda; ^4^Makerere University, Department of Plant Sciences, Microbiology and Biotechnology, P.O. Box 7062, Kampala, Uganda; ^5^Makerere University, College of Health Sciences, School of Medicine, Clinical Epidemiology Unit, P.O. Box 7072, Kampala, Uganda; ^6^National Crops Resource Research Institute, P.O. Box 7084, Kampala, Uganda; ^7^Mbarara University of Science and Technology, Faculty of Medicine, Department of Pharmacy, P.O. Box 1410, Mbarara, Uganda

## Abstract

**Introduction:**

*Mangifera indica* leaves are among the most common materials employed in manufacturing herbal medicinal products. Despite the phytochemical variation of *M. indica* cultivars, there are no monographs to guide the cultivation, processing, and authentication of the materials.

**Methods:**

This study characterized 15 Ugandan *M. indica* leaf varieties, with reference to extraction index (EI), total phenolic content (TPC), antioxidant activity (AOA), and mangiferin concentration (MC). In addition, HPLC fingerprints were established to evaluate the overall phytoequivalence of the materials. Then, using hierarchical clustering (HC) and principal component analysis (PCA), the materials were assigned quality grades.

**Results:**

The mean EI was 9.39 ± 1.64% and varied among the varieties (*P*=0.001); the TPC varied significantly (*P* < 0.0001), from 183.29 ± 2.36 mg/g (Takataka) to 79.47 ± 0.58 mg/g (Apple mango). AOA ranged from 16.81 ± 2.85 *μ*g/mL (Doodo red) to 87.85 *μ*g/mL (Asante). MC varied significantly (*P* < 0.0001), from 105.75 ± 0.60 mg/g (Kate) to 39.53 ± 0.30 mg/g (Asante). HC gave four major grades: A to D (A, varieties with the highest TPC, MC, and AOA). These parameters reduced to below average from group B to group D. The chromatographic fingerprints were visually similar, but the number of peaks varied, from 19 (Kawanda green) to 29 (Kawanda wide), with 23.5 ± 2.9 average peaks. Whole fingerprints were less similar (*r* < 0.8) than common peak fingerprints (*r* > 0.9, *P* < 0.001). PCA grouped the fingerprints into five clusters; loading plots for PC 1 and 2 revealed two important compounds, one at Rt = 15.828 minutes (mangiferin) and the other at 6.021 minutes. Using the standardized common fingerprints, unknown field samples clustered closely with Koona, Kate, and Kawanda green varieties.

**Conclusions:**

The EI, TPC, MC, and AOA values can be utilized to monitor consistency in the quality of materials and the production process. The grades generated can be used to select materials for cultivation and manufacturing. Where minimum concentrations are set, materials of different concentrations are used to dilute or concentrate each other. The HPLC fingerprints can be utilized to authenticate the materials. More samples from different agroecological regions of the country should be tested to cater to climatic variations in order to develop GMP-compliant botanical identification methods.

## 1. Introduction

*Mangifera indica* L. is one of the most common plants in the tropical world. It is primarily grown for its delicious and nutritious fruits [1]. The fruits are considered a good source of essential amino acids such as valine, methionine, cysteine, and isoleucine; vitamins A and C; minerals including calcium, magnesium, zinc, and iron; carotenoids especially *β*-carotene; sugars maltose, glucose, and fructose; and dietary fiber [2]. Communities all over the world use different parts of the *M. indica* including the stembark, leaves, seed kernels, fruits, roots, and flowers which are used to treat different ailments [3]. Furthermore, several studies have demonstrated various bioactivities of *M. indica* such as antimicrobial, antitumor, antidiabetic, anti-inflammatory, antiallergic, and immunomodulatory effects [3, 4]. One of the major mechanisms of action of *M. indica* extracts is through amelioration of oxidative stress, which arises due to the failure of the body to detoxify reactive oxygen species (ROS) and free radicals. These compounds react with body components that have electron-rich functional groups such as proteins, lipids, and DNA. This leads to changes in their structure and function and development of diseases including diabetes, cardiac damage, renal failure, hepatotoxicity, and cancers [5]. The generation of ROS is promoted by factors such as ionizing radiation, chemical pollutants, heavy metals, and drugs. Antioxidants work by sacrificial reaction with ROS to produce neutral unreactive oxygen products and/or by chelating heavy metal ions, to reduce the generation of free radicals [6]. The most important antioxidants in *M. indica* extracts include ascorbic acid, carotenoids, and phenolic components. Of equal importance are the minerals: copper, zinc, manganese, and iron, which are cofactors of enzymes relevant in ROS detoxification cascade [4]. While carotenoids and vitamin C are high in *M. indica* fruit, the most important antioxidant phytochemicals in the stembark and leaves are the phenolic compounds [7]. These include tannin derivatives such as protocatechuic acid and gallic acid, flavonoids such as quercetin, catechin, and kaempferol, and xanthones such as mangiferin. The antioxidant activities of phenolic compounds have been shown to exhibit secondary protective effects against a number of chronic disorders including carcinogenicity, hepatotoxicity, cardiotoxicity, and diabetes. Among the most studied phenolic compounds from *M. indica* are mangiferin and its derivatives. Mangiferin exhibits several pharmacological activities such as antimicrobial [8, 9] and immunomodulatory [10, 11]. In addition, mangiferin also has protective effects on hepatic, cardiac, renal, and brain tissues against induced oxidative stress [12, 13] and inhibits carcinogenesis [14, 15].

Several studies have indicated that the phenolic content of *M. indica* leaf extracts varies greatly with the part of the plant [16], variety [17, 18], climatic conditions at the cultivation site, and agricultural practices [7]. Therefore, pharmaceutical *M. indica* raw materials need to be standardized to ensure consistency in quality of the herbal products. In Uganda, *M. indica* stembark and leaf materials are widely employed in manufacturing products indicated for treatment of respiratory tract disorders including whooping cough, catarrh, sore throat, congestion from asthma, and bronchitis [19]. Various cough syrups containing the plant have been authorized for marketing by the National Drug Authority [19, 20].

*M. indica* materials are sourced either from the wild or from tree fruit plantations. Despite the fact that over 16% of the registered herbal products in Uganda contain materials from *M. indica*, there are no local monographs to guide the cultivation, processing, identification, and chemical characterization of the materials. Still, *M. indica* materials are not included in the readily available WHO monographs or the West African and African pharmacopoeias [19]. The development of a botanical identification method compliant to current good manufacturing practices (cGMP) requires the establishment of chemical profiles of the materials accompanied by chemometric databases [21]. This study developed two criteria: (i) grades of leaves based on quantity of selected phytochemical parameters and (ii) similarity of HPLC fingerprints, which can be used to select sources of the *M. indica* raw materials, authenticate them, and control extraction processes to ensure consistency in the quality of products. The phytochemical parameters chosen for grading of the *Mangifera indica* cultivars included extraction index, mangiferin concentration, antioxidant activity, and total phenolic content. These parameters were particularly chosen because of their relevance to biological activity of the plant as outlined above. In addition, the use of extractable matter as a quality control method is recommended by WHO especially for plants without a suitable chemical or biological assay method [22].

## 2. Methods

### 2.1. Study Design

This was an exploratory experimental study to establish the phytochemical relationships among Ugandan *M. indica* cultivars growing at the National Crops Resources Research Institute (NaCRRI). These cultivars were purposely bred in 2007 to increase fruit yield and have since been distributed to farmers countrywide [23]. These are Apple mango, Sejjembe, Kawanda green, Kate, Asante, Kawanda wide, Suu, Koona, Kagoogwa, MPI, Takataka, Boribo, Ngoogwe, Bire, and Doodo red [23]. To characterize the varieties, we determined their extractive indices (%yields), total phenolic contents, antioxidant activities, and mangiferin concentrations. We then used these data to classify the cultivars into different pharmaceutical raw material grades with the aid of chemometric techniques. Furthermore, HPLC fingerprints were established to evaluate the overall phytoequivalence of the leaf varieties.

### 2.2. Study Area

The NaCRRI was established in Namulonge village, Wakiso District, 19 km north of Kampala, Uganda. Namulonge lies at 1150 metres above sea level, within the agroecological zone of Lake Victoria Crescent at GPS coordinates 0.5288°N, 32.6123°32.6123°E. It is characterized with a mean daily temperature of 20.8°C, 258.89 millimetres of rainfall annually, and red sandy clay loam soils of pH 4.9-5.0. These climatic conditions favor the proliferation of *M. indica* [23]. Field samples were collected from different districts around Uganda.

### 2.3. Collection and Processing of Plant Materials

Mature leaves (about 2 kg) of all the *M. indica* cultivars were collected from NaCRRI in June 2022, with the aid of a taxonomist. Field samples were picked from different districts of the country. Samples were picked from three different trees. The samples were packed in polythene bags, labelled and transported to the Pharmaceutical Chemistry Laboratory, at the Department of Pharmacy at Makerere University for further processing. The fresh samples were washed under running tap water and dried under shade for two weeks. The dried materials were ground into fine powders using an electric grinder and composite samples constituted for each variety. About 30 g of leaf powder for each cultivar was macerated in 150 ml of 70% v/v ethanol in darkness for 4 days at room temperature with occasional shaking. This was done in duplicate. The extracts were filtered using Whatman No. 1 filter paper, and the solvent was evaporated to dryness in a rotary evaporator [24, 25].

### 2.4. Chemicals

Mangiferin standard, methanol, ethanol, orthophosphoric acid, Folin–Ciocalteu reagent, DPPH, gallic acid, sodium bicarbonate, and ascorbic acid were of analytical grade and obtained from Kobian Scientific Uganda, a local agent for Sigma Aldrich® Germany. Distilled and deionized water was prepared in our laboratory.

### 2.5. Determination of the Ethanolic Extraction Index of *M. indica* Leaves

About 30 g of leaf powder for each cultivar was macerated in 150 ml of 70% v/v ethanol in darkness for 4 days at room temperature with occasional shaking. The extracts were filtered using a filter paper, and the solvent was evaporated to dryness using an oven. The extraction index was calculated as the percentage yield following extraction of the plant materials according to the following equation:

(1) $$\begin{array}{c} extraction\;index=\left(\frac{Mr}{Ms}\right)\times 100\text{,} \end{array}$$

where Mr denotes the mass of the dried residue and Ms denotes the mass of the extracted leaf powder [22].

### 2.6. Determination of Total Phenolic Content of the *M. indica* Leaf Extracts

The total phenolic content (TPC) of the extracts was determined using the Folin–Ciocalteu method as applied in [26]. The sample powders (100 mg) were dissolved in 10 mL of distilled water. A volume of 0.5 mL of the test solutions was transferred into vials, and then 0.5 mL of Folin–Ciocalteu reagent was added. After about 10 minutes, 1.5 mL of 2% (w/v) sodium carbonate solution and 4.5 mL of distilled water were added. The reaction mixture was incubated in the dark at room temperature (28°C) for 30 minutes. The TPC of the samples was determined from their visible electromagnetic energy absorbances at 755 nm, in comparison to gallic acid standard solutions using a UV/visible spectrophotometer (Jenway 6705 UV/Vis, Bibby Scientific, United Kingdom).

### 2.7. Determination of Antioxidant Activity

The antioxidant activity was determined using the DPPH scavenging activity as applied in [27]. Sample solutions were prepared by dissolving the powdered leaf materials (0.1 g) in 10 mL of 99.9% methanol, by orbital shaking at 200 rpm for 30 minutes. The solutions were then filtered and made up to 10 mL with methanol. To prepare the standard solutions of ascorbic acid, 0.01 g was dissolved in 10 ml of 99.9% methanol; then, 1 mL of this solution was further diluted to 10 mL with methanol. To determine the scavenging activity, 20, 30, 40, and 50 *μ*L of ascorbic acid solutions were each added to a solution of 3 mL DPPH (0.0039 mg/mL) and 1 mL methanol and shaken to mix. The absorbance of each solution was obtained at 517 nm. Methanol was used as the negative control while DPPH (0.0039 mg/mL) solution was the blank. The activity of the test solutions was determined similarly by adding 20, 30, 40, and 50 *μ*L of each sample solution to a solution containing 3 ml of DPPH and 1 mL of methanol. The decrease in absorbance of the ascorbic acid and samples was calculated in comparison to a blank sample containing only methanol and the DPPH.

The percentage decrease in absorbance (hereby referred to as the percentage inhibition) was calculated according to the following equation:

(2) $$\begin{array}{c} \%\;inhibition=\left(\frac{Abs\;blank-Abs\;S}{Abs\;blank}\right)*100\text{,} \end{array}$$

where Abs blank denotes the absorbance of the blank sample and Abs *S* denotes the absorbance of either the test solution or standard ascorbic acid solutions. The % inhibition data were plotted against concentration to determine the amount of the sample and ascorbic that inhibits 50% of the DPPH (IC_50_), the measure of antioxidant capacity.

### 2.8. HPLC Fingerprinting and Quantification of Mangiferin

#### 2.8.1. HPLC System

HPLC analysis was performed on a UPLC Prominence Shimadzu chromatograph (Japan). The HPLC machine comprised a communicator (CBM-20A), UV/visible detector (SPD-20A), SIL-20AC HT autosampler, LC 20 AD pumps, column oven (CTO-20AC), a Phenomenex Luna C18 column (250 cm long, 4.6 mm internal diameter, and 5 *μ*m particle size), and an online degassing unit (DGU-20A).

#### 2.8.2. Preparation of Standard Solutions and Test Solutions

*Mangifera indica* solutions were prepared by dissolving the powdered leaf material in methanol to make 1 mg/mL solutions with sonication. Standard solutions containing 40, 60, 80, 100, 120, 140, and 160 *μ*g/mL were prepared by dissolving mangiferin in methanol and diluting serially.

#### 2.8.3. Mobile Phase Solutions and HPLC Conditions

To obtain the mobile phase solutions, we experimented with solvent fractions developed earlier for quantification of mangiferin and other phenolic compounds [28–30]. The final solvent system composed of methanol (31%) and 0.01% orthophosphoric acid (69%). The optimal mobile phase flow rate was 1 mL/min, while the column temperature was 25°C.

#### 2.8.4. Fingerprint Development, Visualization, and Identification of Markers

Fingerprints were developed by injecting 10 *μ*L of the sample solutions and varying mobile solvent phase systems (isocratic elution) into the chromatograph and visualizing with a UV detector at 258 nm. Mangiferin was identified based on its retention time. The stability of the extracts solutions was assessed by computing the similarity indices (*c* and *r*) of the fingerprints of a sample solution stored for up to three days.

#### 2.8.5. Quantification of Mangiferin in the *Mangifera indica* Test Solutions

The concentration of mangiferin was based on standard calibration curves and the corresponding peak areas. The peaks due to mangiferin were identified after spiking the samples three times; the average retention times were computed and used to identify the markers in the rest of the samples.

#### 2.8.6. Validation of HPLC Quantitative Methods

The accuracy of the method was determined by computing the percentage recovery of mangiferin from three spiked samples. The intraday repeatability and interday repeatability were obtained from the percentage relative standard deviation of three different samples at different concentration levels on the same day and after three days, respectively. The linearity was obtained from the regression equation of the standard calibration curve. The limit of detection (LOD) and the limit of quantification (LOQ) of mangiferin were calculated as 3.3 *∗* SD/slope and 10 *∗* SD/slope, respectively. We assessed peak purity by spiking samples; selective increment in mangiferin *r* peak areas and heights indicated peak purity.

## 3. Data Analysis

Data were captured, stored, and cleaned in Microsoft Excel 2019®. The variation of quantitative data including extraction index, total phenolic content, mangiferin concentration, and antioxidant activity among leaf varieties was analysed by one-way ANOVA, followed by Tukey's multiple comparison tests in GraphPad Prism 9® and Minitab 19® at the confidence levels of 95%. With classical hierarchical cluster analysis of these data, we generated different groups, based on Euclidean distances (PAST 4®); we then used these clusters to group the *M. indica leaf* materials into pharmaceutical grades corresponding to levels of extraction index, total phenolic content, mangiferin concentration, and antioxidant activity.

The HPLC fingerprints were qualitatively analysed by visualization and semi-quantitatively analysed by computing similarity indices. Fingerprints with the best resolution of the components indicated by the number of peaks and peak symmetry were identified. For fingerprint similarity analysis, whole fingerprints (including all peaks) and common fingerprints (with peaks that were common in all samples according to retention time) were identified. Whole fingerprints were compared visually and by calculating similarity indices based on peak areas. For common fingerprints, the relative retention times of the common peaks in reference to mangiferin were computed. Furthermore, fingerprint peaks were compared by similarity indices (correlation coefficient (*r* > 0.9) at *P*=0.01) and principal component analysis (PCA) using PAST 4® software. For PCA, similarity was evaluated by the minimum spanning tree distances in a scatter plot of the first two components [30, 31]. Additionally, loading plots demonstrated the peaks responsible for the variation of fingerprints.

## 4. Results and Discussion

This work aimed at establishing standards for classification of *Mangifera indica* leaf materials obtained from different varieties. To classify the materials, selected parameters relevant to the management of respiratory disorders were quantified and analysed by chemometric techniques to generate different quality grades. *M. indica* extracts alleviate symptoms of respiratory tract disorders by reducing inflammation of the airways and chelating and neutralizing harmful substances, thereby reducing irritation and damage of the respiratory mucosa as well as regulating immune responses. These activities have been demonstrated in *M. indica* extracts rich in phenolic compounds, and particularly mangiferin. Since the antioxidant activity results from the combined effects of many compounds in addition to phenolics, including vitamins, terpenoids, and minerals, it is logical to consider it as a separate phytochemical parameter. In addition to these parameters, HPLC fingerprints were included to give a general picture of the phytochemical variation and to determine if the *M. indica* leaf varieties are phytoequivalent. The use of chemometric methods to analyse the data enabled generation of distinct patterns (grades) of *M. indica* leaves.

### 4.1. Extraction Index (EI), Total Phenolic Content (TPC), Mangiferin Concentration (MC), and Antioxidant Activity (AOA) of *Mangifera indica* Leaves

The mean EI was 9.39 ± 1.64%, and it varied significantly among the varieties (*P*=0.001). Similarly, the TPC and the MC varied significantly (*P* < 0.0001) among the leaf extracts. The TPC ranged from 183.29 ± 2.36 mg/g in Takataka variety to 79.47 ± 0.58 mg/g in Apple mango variety, with an average value of 131.22 ± 32.03 mg/g. The MC was highest in Kate variety (105.75 ± 0.60 mg/g) and lowest in Asante (39.53 ± 0.30 mg/g), with an average of 59.19 ± 18.09 mg/g. The highest AOA was 16.81 ± 2.85 *μ*g/mL for Doodo red while the lowest was 67.67 ± 20.19 *μ*g/mL for Asante; the average was 35.98 ± 18.31 *μ*g/mL. The results are summarized in Table 1, and in the following, each parameter is explained in detail.

### 4.2. Extraction Index (EI)

The extraction index determines the non-structural proportion of the drug biomass that is extracted by solvents. Thus, the extractable matter contains primary metabolites including proteins, lipids, and carbohydrates and their building units and secondary metabolites such as waxes, terpenes, gums, resins, phenolics, alkaloids, essential oils, and inorganic compounds [32]. For native extracts (for which no excipients or other substances are added), the extractable matter is also the final drug, and thus the extraction index exhibits the efficiency of the processing method [33]. Therefore, plant materials with high yields are desirable for profitability of the herbal medicine business. In this study, the EI of *M. indica* leaf materials was highest for Kawanda wide variety at 12.96 ± 0.60% and lowest for MPI variety at 7.74 ± 1.91% (Table 1). These values were similar to those obtained by other researchers [34].

Besides quantification of the drug, WHO recommends the use of extractable matter as a quality control method especially for plants without a suitable chemical or biological assay method [22]. As such, the extraction index can be used to monitor consistency in the quality of raw materials and extraction process or monitor the effect of changes in the manufacturing process and plant source (variety or species). Furthermore, the extract strength is also relevant for calculating of the dosages of the individual materials to include in the product formula [33].

### 4.3. Total Phenolic Content (TPC)

The TPC of *M. indica* is a summation of phenolic acids, flavonoids, and xanthones, the main phytochemicals implicated for antioxidant activity. Therefore, the TPC is an indicator of the quality of materials intended for use as antioxidants or indications based on antioxidant activity: this approach is easier, cheaper, and more relevant than quantifying the individual compounds. The TPC and/or individual phenolic compounds are known to vary among *M. indica* cultivars [17, 18]. In this study, the TPC of the materials varied significantly with the *M. indica* variety (*P* < 0.0001), as demonstrated by Tukey's multiple comparisons (Table S1). Takataka variety had the highest content followed by Kagoogwa while Apple mango and Sejjembe varieties had the lowest (Table 1). The TPC values were similar to those reported earlier [35]. Since there are no established limits for TPC of *M. indica* materials or products for the treatment of respiratory tract disorders, it is incumbent upon the manufacturer to establish the minimum acceptable TPC of raw materials after establishing its relevance to bioactivity (and indication) of their materials and/or products. This can be done by designing dose-response experiments to establish the relationship between the TPC of materials and/product and the ability to ameliorate the symptoms of the disease.

### 4.4. Antioxidant Activity (AOA) of *Mangifera indica* Leaves

Antioxidant activity (AOA) is one of the major biological activities of *M. indica* extract. Actually, some researchers have postulated that many of the other pharmacological activities of the plant are secondary to its ability to scavenge ROS involved in the pathogenesis of the diseases. As such, brain-protective [36], antidiabetic [37], cardio-protective, anti-inflammatory, hepato-protective [38, 39], reno-protective, and anticancer activities [40] have been demonstrated. This implies that a measure of the AOA of the raw materials is a direct measure of their potency and so ensures pharmacological reproducibility [41]. This approach is more appropriate than measuring quantities of individual compounds or groups of compounds (e.g., TPC); besides, it is not cost-effective to determine all the active compounds. In addition, it is more practical for the manufacturer to measure the AOA of the plant materials than determining the therapeutic effect (in this case, several activities relevant to treating respiratory tract disorders) as a quality assurance measure. Given the fact that the composition of antioxidant phytochemicals varies with the variety or plant species, the AOA of *M. indica* leaf materials is expected to vary with the source cultivar. In this study, it varied significantly (*P* < 0.0001) (Table S7). Doodo red and Kate varieties had the highest AOA (lowest IC_50_), i.e., 16.81 ± 2.85 *μ*g/mL and 18.35 ± 1.49 *μ*g/mL, respectively. On the other hand, the AOAs of Asante (IC_50_ = 67.67 ± 20.19 *μ*g/mL) and MPI (IC_50_ = 65.88 ± 8.85 *μ*g/mL) were the lowest (highest IC_50_) (Table 1). The values of AOA obtained in this study are similar to those reported elsewhere [42].

### 4.5. Mangiferin Concentration (MC)

Mangiferin is one of the most studied phenolic compounds of *Mangifera indica* with several pharmacological activities as outlined in the introduction. For the respiratory tract, mangiferin reduces inflammation of the airway, inhibits cytokine production, and protects against lipopolysaccharide-induced allergy [43, 44]. This makes it a favorable candidate for use as an activity marker [45]. In addition to a diverse biological profile, mangiferin is found in only a few other plant species such as *Iris unguicularis*, *Anemarrhena asphodeloides*, *Bombax ceiba*, *Salacia* sp., *Cyclopia* sp., and *Crocus* sp. [46], which are morphologically distinct from *M. indica.* This makes it ideal as a bioanalytical marker. Besides, the analytical standard is readily available commercially and can be easily isolated in high amounts from several parts of the plant using common solvents like methanol and ethanol. In addition, mangiferin can be quantified by basic spectroscopic and HPLC methods (the method used in this study). For use as pharmaceutical raw materials, it is only logical that cultivars with high mangiferin concentrations are desirable. In this study, Kate variety had the highest mangiferin concentration, followed by Koona and Ngoogwe while Asante, Apple mango, and Kawanda wide had the lowest (Table 1). The MC varied significantly with *M. indica* variety (*P* < 0.0001), as demonstrated by ordinary one-way ANOVA and Tukey's multiple comparisons (Table S3).

### 4.6. Relationships among the Phytochemical Parameters

The Pearson correlation analysis of the parameters showed a direct (positive) relationship, as expected, with the correlation between the mangiferin concentration and antioxidant activity being statistically significant at *α* = 0.05 (Table 2).

The TPC positively correlated with the extraction index, although not significantly (*r* = 0.443, *P*=0.098). In addition, the TPC/EI ratio, an indicator of how much of the extracted matter is active drug (where activity due to phenolic compounds is of primary interest), ranged between 8.0 and 18.4 with a mean of 14.2. Koona, Kawanda green, and Takataka varieties had the highest TPC for a unit percentage yield of the extract, while Apple mango, Doodo red, and Sejjembe had the lowest (Table S1). This observation can be explained by the fact 70% ethanol extracts a variety of relatively polar compounds besides phenolic compounds, the concentration of which could also vary in different varieties. Some of these compounds, like terpenoids and minerals, augment the bioactivity of phenolics and so are desirable [4].

**Figure 1:**
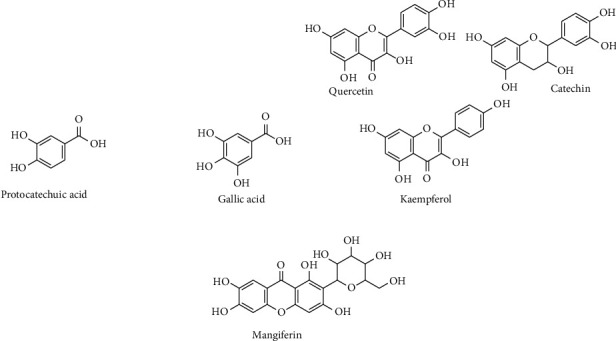
Examples of phenolic compounds in *Mangifera indica* leaves, showing different kinds of the phenolic nucleus, number, and distribution of hydroxyl group. (a) Phenolic acids, (b) Flavonoids, and (c) Xanthones. The structures were drawn by ChemBioDraw Ultra® version 14.

A correlation analysis revealed that the AOA of *M. indica* materials increases proportionately with increase in the TPC although not statistically significant (*r* = −0.292, *P*=0.291). Actually, some samples with high TPC had low antioxidant activity such as Asante, Kawanda green, and Kawanda wide. This notwithstanding, most varieties showed a direct relationship between the TPC and AOA, that is, Kate, Koona, Kagoogwa, and Takataka. Some varieties such as Doodo red had comparatively high AOA despite lower TPC (Table 1). The low correlation between TPC and AOA can be explained by the fact that *M. indica* contains (i) other non-phenolic antioxidant compounds such as terpenoids, carotenoids, vitamins E and C, and minerals and (ii) phenolic compounds with low or no AOA such as amino acids [7, 16]. However, these components are known to concentrate mainly in the fruits. Their role as antioxidants in other parts of the plant is yet to be elaborated; (iii) another factor is the variation in the concentration of the specific phenolic compounds with varying activity. Structure activity relationship analysis of different phenolic compounds indicates that antioxidant activity is affected by the number of aromatic and hydroxyl groups (Figure 1) as well as their relative positions in the structure [47], and thus tannins (A), flavonoids (B), and xanthones (C) exhibit different levels of activity.

Thus a *M. indica* variety may only produce a low of TPC but contain a high concentration of the compound(s) with high activity and vice versa. In addition, some phenolic compounds might work additively, synergistically, or antagonistically as demonstrated in [48]. While the correlation between the TPC and AOA was low, the AOA/TPC ratio should be established and utilized to monitor the consistency in the composition of the herbal materials. The AOA/TPC ranged from 1.3 to 8.9 with an average of 4.2 (Figure S1).

There was a significant correlation between the mangiferin concentration (MC) of the *Mangifera indica* leaves and their antioxidant activity (AOA) (*r* = −0.567, *P* = 0.028). This shows, as expected, that the AOA increases (reducing IC_50_) with an increase in the MC. This correlation was higher than that seen with TPC and AOA. The results demonstrate the importance of mangiferin as an antioxidant component of *M. indica* leaves, qualifying it as a marker for AOA. Since *r* < 1, this validates the fact that the observed total AOA is a result of synergism among the various phytochemicals. Actually, some samples with relatively lower MC such as Takataka had relatively high AOA; such samples are likely to be rich in non-mangiferin antioxidant compounds. The AOA/MC ratios ranged from 1.2 to 6.9, with an average value of 2.8 (Table S1). The manufacturer can set a minimum acceptable ratio depending on the relevance of mangiferin to the application of the materials or products.

Generally, there was a positive although not significant correlation between the mangiferin concentration (MC) of the *M. indica* leaves and the total phenolic content (TPC) (*r* = 0.363, *P*=0.184). Samples with the highest MC per TPC were Ngoogwe, Bire, and Kate while Asante, Kawanda wide, and Kawanda green had the lowest. The MC/TPC ranged from 0.3 to 0.6, with a mean of 0.5. These results show that mangiferin is just one of the phenolic compounds in *M. indica*.

### 4.7. Classification of the *Mangifera indica* Leaf Varieties Based on Extraction Index, Total Phenolic Content, Mangiferin Concentration, and Antioxidant Activity

#### 4.7.1. Clustering Analysis

**Figure 2:**
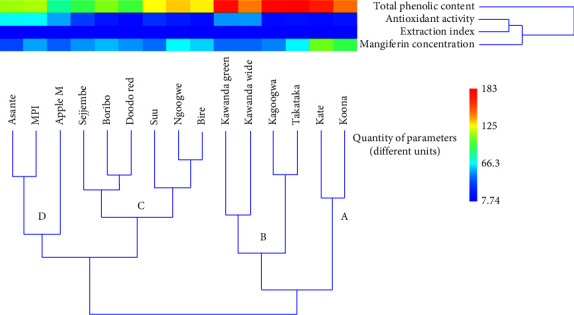
Dendrogram for the classical hierarchical cluster analysis of *M. indica* leaf varieties. Algorithm: Ward's method; similarity index, Euclidean distance.

Clustering is a multivariate analysis tool that groups samples based on the similarity of the measured parameters. In this study, we used hierarchical clustering to classify *Mangifera indica* leaf cultivars depending on the variation of four parameters, namely,extraction index, total phenolic content, mangiferin concentration, and antioxidant activity. The similarities were performed using Ward's method algorithm. Four main groups, A, B, C, and D, were obtained (Figure 2).

Group A contains varieties with the highest total phenolic contents, mangiferin concentrations, and antioxidant activities. These parameters reduce from group B to group D, to below average values. Based on these clusters and on the average quantities of the studied parameters, we generated four grades of *M. indica* leaf materials. These are summarized in Table 3.

**Figure 3:**
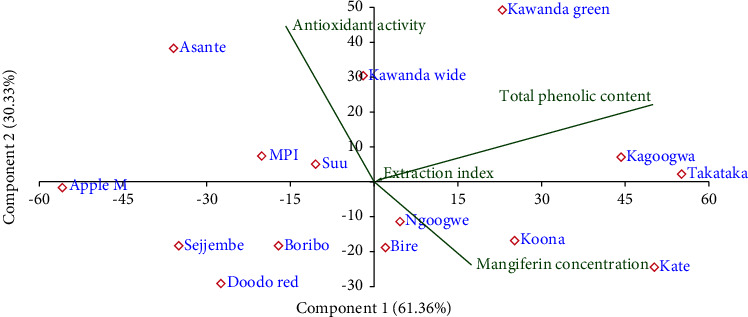
PCA scatter biplot of various *Mangifera indica leaf* cultivars showing contribution to variation of different phytochemical parameters; factor scores of observations were plotted on the first two components (drawn by PAST software version 4.12).

The contribution of the parameters to the observed clusters is well illustrated by a PCA scatter biplot (Figure 3).

From Figures 2 and 3, it is clear that the total phenolic content varies most, followed by the mangiferin concentration and antioxidant activity. The extraction index is the least variable parameter. These grades generated can guide manufacturers and botanists to select the best varieties for use as pharmaceutical raw materials. For therapeutic applications for which the studied parameters are relevant, such as respiratory tract disorders (as it is in Uganda), samples with high parameters are preferred. However, the manufacturer might need to determine the minimum amounts of each parameter that provides optimum potency of the product; this was beyond the scope of this work.

#### 4.7.2. Classification of the *Mangifera indica* Leaf Varieties Based on Fingerprint Characteristics

**Figure 4:**
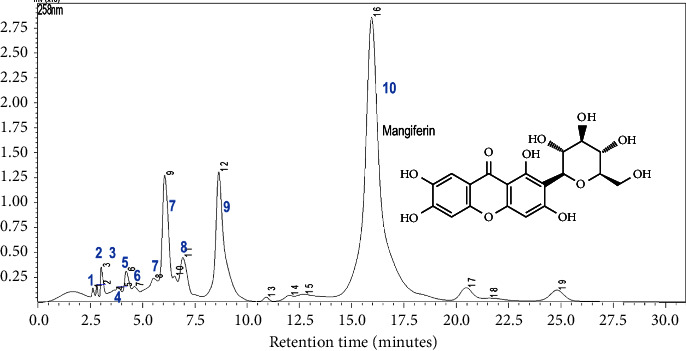
Representative HPLC fingerprint of *M. indica* leaf ethanolic extract showing common peaks (blue numbers).

The classification of *Mangifera indica* leaf varieties is based on the common fingerprint pattern recognition and multivariate analysis of common peak (common fingerprints) and whole chromatogram peak areas (whole fingerprint). The typical fingerprint is shown in Figure 4.

#### 4.7.3. Visual Analysis for Pattern Recognition of HPLC Fingerprints

**Figure 5:**
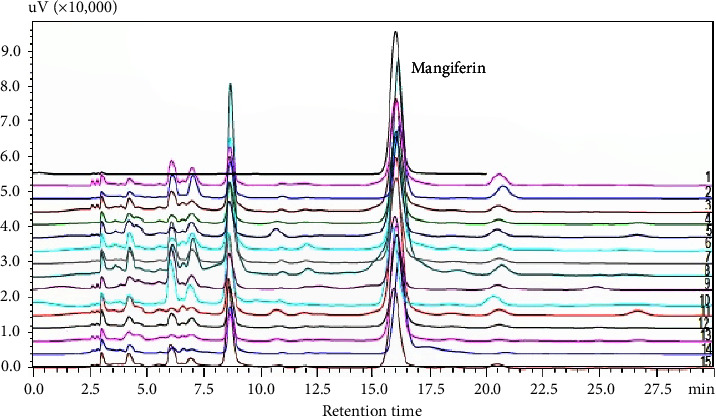
HPLC fingerprint overlay showing the similarities of the different varieties of *Mangifera indica* leaf extracts. 1, Apple mango; 2, Asante; 3, Bire; 4, Boribo; 5, Doodo red; 6, Takataka; 7, Kagoogwa; 8, Kate; 9, Kawanda green; 10, Kawanda wide; 11, Koona; 12, MPI; 13, Ngoogwe; 14, Sejjembe; 15, Suu.

Visual inspection of the 30 minutes of whole fingerprints showed high similarity (Figure 5). However, individual fingerprints varied greatly in the number of peaks, from 19 in Kawanda green to 29 in Kawanda wide, with an average of 23.5 ± 2.9 peaks. The total peak areas ranged from 5,863,448 mVmins for the Kate variety to 1,568,633 mVmins for the Asante variety, with an average area of 2,457,451 ± 1,026,790 mVmins (Table S5).

These results demonstrate marked phytochemical variability of the *M. indica* varieties. This can be explained by the fact that the genetic makeup of plants determines the nature and amounts of plant metabolites by influencing the nature and number of enzymes and cofactors produced by a particular cultivar or subspecies [49]. Also, certain cultivation of plants in non-natural habitats may affect their metabolic rate because of unfavorable climatic conditions in the new environments [50]. Thus, a project whose aim is to domesticate medicinal plants needs prior investigation of the suitability of the agroecological factors in the new habitat. In absence of specific markers, whole fingerprints can be used to demonstrate similarity phytoequivalence of medicinal plant varieties and also study the effect of changes in cultivation, harvesting, and postharvest handling practices [51].

To reduce the complexity and cost of analysing many markers in plant materials, the Chinese pharmacopoeia recommends analysis of common fingerprints, which are constructed peaks that are common to all samples (same retention times) [52]. Common fingerprints are also easier to reproduce than whole fingerprints. For this study, we obtained ten common peaks (Figure4); their retention times and peak areas are shown in Table S4. The variety “Kawanda wide,” which had the highest number of peaks, was used as the reference in selection and matching of peaks. The total area of common fingerprint peaks ranged from 3,710,796 mVmins to 961,454 mVmins with an average of 1,652,214 ± 652910 mVmins. The variations in peak areas are proportional to the variations in concentration of the compounds responsible for the peaks and thus show the variability of the samples. The pattern of the peaks is characteristic of the plant material for the specified analysis conditions and so can be used to identify and authenticate the materials. The fingerprints we developed were reproducible (Figures S1 and S2). In absence of markers or if the chemical composition of the material is not known, strong peaks (peak area more than 10% of the total area) are used as reference in describing relative positions and areas of other peaks [53]. For this study, only peak 10 (mangiferin, Rt = 15.828 mins) was a strong peak, making up more than 70% of total peak area (Table S5).

#### 4.7.4. Fingerprint Similarity Analysis of Fingerprints Using Correlation Indices

Besides visual and descriptive evaluation, fingerprints can also be compared for similarity by calculating similarity indices such as correlation coefficients (*r*), cosines (*c*), and Euclidean distances (ED), among others. We calculated *r* of the whole and common fingerprints to determine the similarity of the leaves from different *M. indica* varieties (note: the whole fingerprints consist of all peaks in the chromatogram of each samples, while the common fingerprints constitute only peaks that are “common” to all the chromatograms of different samples).

**Figure 6:**
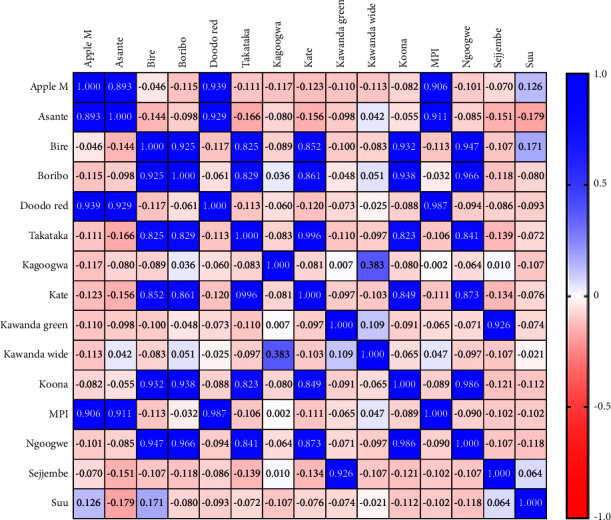
Heat map of the Pearson correlation matrix of *Mangifera indica* leaf variety fingerprints based on whole chromatogram peak areas; the intensity of blue is proportional to the degree of similarity of the fingerprints.

The whole fingerprints showed low correlation, with only a few fingerprints having *r* > 0.8 (Figure 6).

**Figure 7:**
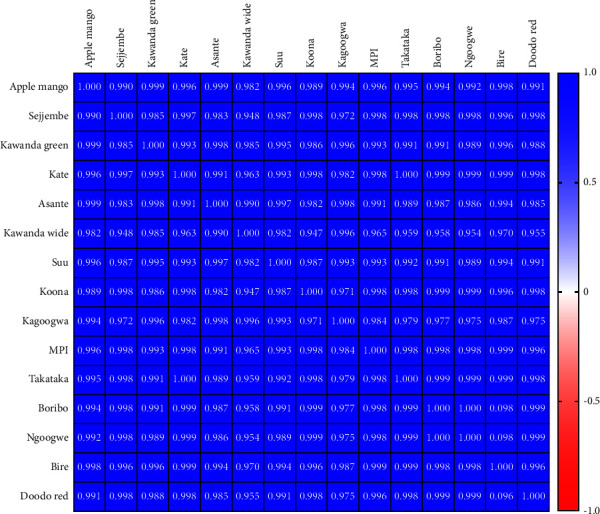
Heat map of the Pearson correlation matrix of *Mangifera indica* leaf variety fingerprints based on common peak areas; the intensity of blue is proportional to the degree of similarity of the fingerprint (drawn by GraphPad Prism version 9).

On the other hand, the Pearson correlation analysis of common fingerprints showed that all the *Mangifera indica* leaf varieties were significantly similar (*r* > 0.9, *P* < 0.001), as shown in Figure 7.

This information can be used to guide the manufacturer in selecting phytoequivalent materials. According to the Chinese pharmacopoeia, only samples with *r* ≥ 0.9 are considered identical and therefore phytoequivalent; such materials can be substituted without significantly altering the chemical composition and thus the potency of product [52]. This approach is more accurate than analysing just a few markers [54], Hence, from Figure 6, the following varieties are equivalent: Apple mango = Doodo red = MPI; Asante = MPI = Doodo red; Bire = Boribo = Doodo red = Koona = Ngoogwe; Takataka = Kate; Kawanda green = Sejjembe; Kawanda wide and Suu varieties have no substitutes. Although common fingerprint analysis gives higher *r* values (Figure 7), these fingerprints are based on only a few compounds. Therefore, it is crucial to study the plant material extensively to ensure that the selected peaks represent the most important active phytochemicals, in order to generate an accurate bioactivity fingerprint [31]. Nevertheless, common peak fingerprints are valuable in authenticating herbal raw materials or products [55].

#### 4.7.5. Principal Component Analysis (PCA) of Common Fingerprints

**Figure 8:**
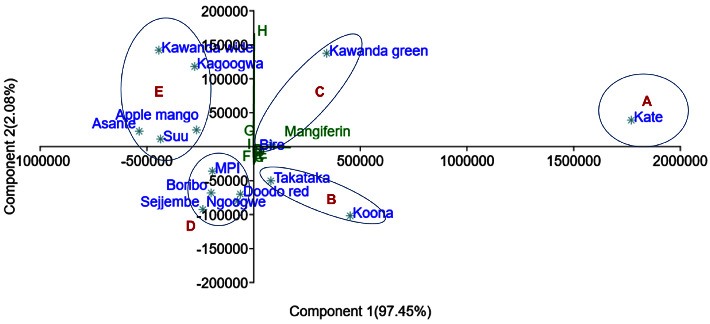
PCA scatter biplot of *Mangifera indica* leaf fingerprints and the contributing peaks; factor scores of observations were plotted on the first two components (drawn by PAST software version 4.12).

**Figure 9:**
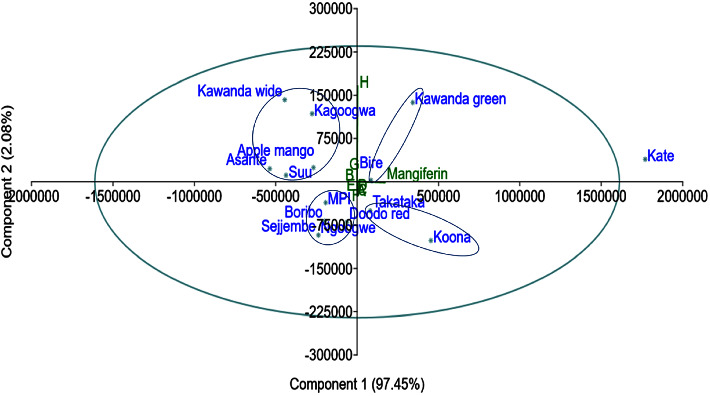
PCA scatter biplot of *Mangifera indica* leaf fingerprints and the contributing peaks; the 95% confidence ellipse showing the Kate variety as an outlier (drawn by PAST software version 4.12).

In the PCA scatter plot, all the *Mangifera indica* leaf varieties lie within the 95% ellipse apart from Kate (Figures 8 and 9).

**Figure 10:**
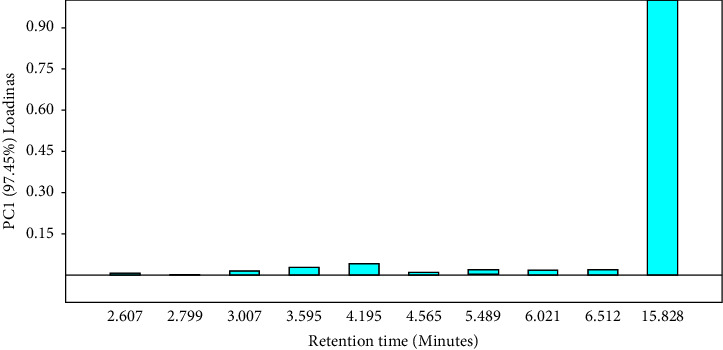
Loading plots of *Mangifera indica* leaf fingerprints showing the contribution of the peaks to the variance of PC1 (97.45%); peak 10 (mangiferin) (Rt = 15.828 minutes) contributed the most to PC1 variance (loading score >0.9) (drawn by PAST software version 4.12).

**Figure 11:**
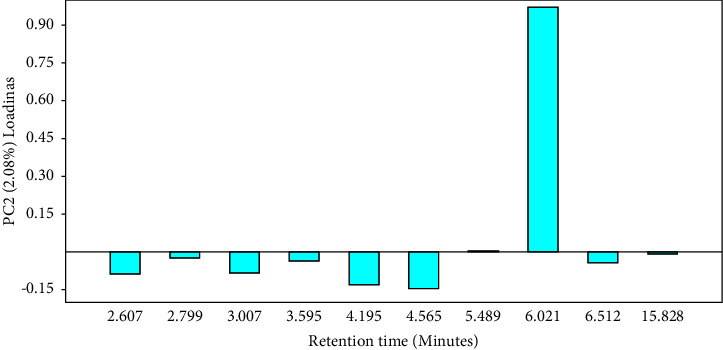
Loading plots of *Mangifera indica* leaf fingerprints showing the contribution of the peaks to the variance of PC2 (2.08%); peak 8 (Rt = 6.021 minutes) contributed the most to the variance of PC2 (loading score >0.9) (drawn by PAST software version 4.12).

Since it is not undesirable to have high a concentration of mangiferin, we did not eliminate Kate variety from the classification but rather assigned it as a separate group. Thus, from Figure 8, five major groups are noticeable: A (Kate), B (Koona and Takataka), C (Bire, and Kawanda green), D (MPI, Doodo red, Boribo, Sejjembe, and Ngoogwe), and E (Asante, Suu, Kawanda wide, Kagoogwa, and Apple mango). These arise due to the variations in the concentration of the chemical constituents of the samples, as illustrated by the biplot (in green) and by Figures 10 and 11. Here, the loading plots for principal components 1 and 2 revealed that most of the sample variance is caused by two compounds, one at Rt = 15.828 minutes (mangiferin) and the other at 6.021 minutes (compound 8 (H)).

Thus, in reference to Figure 8, groups A and B consist of varieties with the highest mangiferin concentration but above average concentration of compound 8 (H); varieties in Group C have above average mangiferin and compound 8 concentrations; group D varieties have average mangiferin concentration and low compound 8 concentration, while group E varieties have the lowest mangiferin concentration and above average compound 8 concentration.

#### 4.7.6. Comparison of the HPLC Fingerprints of the *M. indica* Cultivars with Those of Unidentified Samples Collected from Various Parts of the Country (Field Samples)

**Figure 12:**
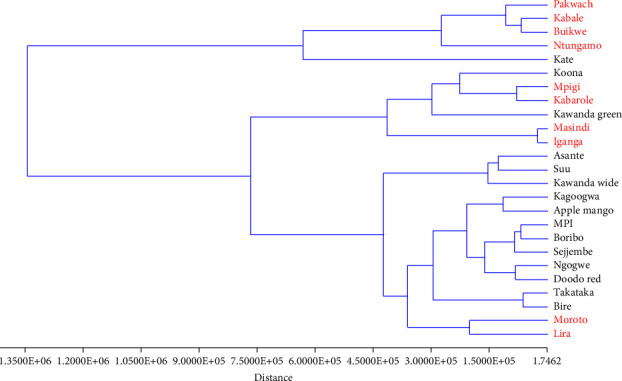
Dendrogram for the classical hierarchical cluster analysis of *M. indica* leaf varieties and field samples. Algorithm: Ward's method; similarity index, Euclidean distance. The labels in red indicate the samples collected from different districts around Uganda.

To identify the groups of materials to which the field samples relate to, the common fingerprints of unknown samples collected from ten different districts in Uganda were compared to the standardized fingerprints of the 15 cultivars. The results indicated that most of the field samples were close to Koona, Kate, and Kagoogwa green varieties. The others were close to Takataka and Bire varieties as illustrated by Figure 12.

While the actual variety to which the field samples belong can only be confirmed with genetic studies such as DNA bar coding, the results obtained give an insight into the most cultivated *Mangifera indica* varieties and thus the sources of the herbal materials. According to [23], Kate, Koona, Kawanda green, Takataka, and Bire are among the high fruit yielding varieties, hence grown by most households. Coincidentally, Kate, Koona, and Takataka varieties belong to group A (high in TPC, MC, and AOA), according to our grading, while Kawanda green belongs to group B. However, Bire belongs to the lowest group (D). Perhaps more samples are needed to further test the method and validate these results.

## 5. Conclusions

This study has demonstrated that the *Mangifera indica* leaf materials have relatively similar ethanolic extractive indices but differ in the total phenolic content and mangiferin concentration and thus antioxidant activity. Based on these parameters, we graded the raw materials and showed the varieties that can be substituted (those in the same quality grade) for production of medicines for respiratory tract disorders. These parameters and the ratios of their quantities can also be utilized to monitor the consistency in the quality of materials and the production process. In addition to the quality grades, we also developed HPLC fingerprints which can be utilized to authenticate the materials by demonstrating phytoequivalence at correlation coefficients greater than 0.9. For standardization purposes, where minimum required mangiferin marker concentrations are set, materials of different concentrations are used to standardize each other, i.e., dilution or concentration. This approach is preferred to addition of pure markers (e.g., mangiferin) to dilute samples or addition of inactive substances (excipients) to concentrated materials. Also, to develop a GMP-compliant botanical identification method (BIM), both “diluted” and “concentrated” materials are needed. That said, more sampling and testing are necessary to cater for as much variability as possible. There is also a need to test materials growing in different agroecological regions of the country to cater for climatic influence and generalize the application of the analytical parameters.

## Figures and Tables

**Table 1:** Extraction index, total phenolic content, mangiferin concentration, and antioxidant activity of *Mangifera indica* leaves obtained from fifteen different varieties.

No.	*Mangifera indicacta* variety	Mean extraction index (EI) (%)	Mean total phenolic content (TPC) of extract (mg/g GAE)	Mean mangiferin concentration of extract (MC) (mg/g)	Antioxidant activity (AOA) (IC_50_*μ*g/mL)
1	Apple mango	9.90 ± 0.56^ab^	79.47 ± 0.58^m^	44.60 ± 0.34^k^	55.41 ± 2.05^abc^
2	Sejjembe	9.01 ± 0.59^ab^	92.54 ± 0.23^l^	53.52 ± 0.21^g^	37.97 ± 2.55^bcde^
3	Kawanda green	9.45 ± 0.19^ab^	171.74 ± 1.43^c^	46.61 ± 0.07^j^	53.66 ± 22.10^abcd^
4	Kate	11.85 ± 0.00^ab^	163.59 ± 0.21^d^	105.75 ± 0.60^a^	18.35 ± 17.95^e^
5	Asante	8.02 ± 4.39^b^	112.94 ± 0.42^i^	39.53 ± 0.30^l^	67.67 ± 20.19^a^
6	Kawanda wide	12.96 ± 0.42^a^	141.58 ± 0.55^f^	46.36 ± 0.07^j^	53.80 ± 13.58^abcd^
7	Suu	9.85 ± 0.20^ab^	124.63 ± 0.46^h^	47.56 ± 0.67^i^	33.69 ± 10.98^cde^
8	Koona	7.84 ± 0.32^b^	144.33 ± 0.70^e^	92.66 ± 0.18^b^	23.08 ± 9.00^e^
9	Kagoogwa	11.49 ± 0.08^b^	177.13 ± 1.23^b^	51.10 ± 0.15^h^	19.74 ± 3.36^e^
10	MPI	7.74 ± 1.35^b^	114.98 ± 0.40^i^	55.70 ± 0.25^f^	65.88 ± 8.85^ab^
11	Takataka	10.01 ± 0.89^ab^	183.29 ± 2.36^a^	65.55 ± 0.26^c^	22.82 ± 2.26^e^
12	Boribo	7.77 ± 1.58^b^	108.79 ± 0.61^j^	58.30 ± 0.02^e^	25.17 ± 4.30^de^
13	Ngoogwe	8.14 ± 0.76^a^	130.83 ± 0.06^g^	65.94 ± 0.06^c^	26.93 ± 4.60^cde^
14	Bire	7.95 ± 1.76^b^	126.41 ± 0.31^h^	61.40 ± 0.39^d^	18.71 ± 3.18^e^
15	Doodo red	8.88 ± 0.73a^b^	96.06 ± 0.06^d^	53.29 ± 0.27^g^	16.81 ± 2.85^e^
		9.36 ± 1.64	131.22 ± 32.03	59.19 ± 18.09	35.98 ± 18.31

GAE, galic acid equivalent. The mean values of parameters that do not share a letter are significantly different (*α* = 0.05). The AOA of ascorbic acid was 2.94 ± 0.12 *μ*g/mL.

**Table 2:** Pearson correlation for the studied phytochemical parameters.

Parameters	Total phenolic content	Antioxidant activity	Mangiferin content
	*r* (*P* value)	*r* (*P* value)	*r* (*P* value)
Antioxidant activity	−0.292 (0.291)		
Mangiferin content	0.363 (0.184)	−0.567 (0.028)^*∗*^	
Extraction yield	0.443 (0.098)	−0.053 (0.853)	0.043 (0.879)

^*∗*^Significant at the 95% confidence level (*P* < 0.05).

**Table 3:** Classification of *Mangifera indica* leaf varieties based on the extraction index, total phenolic content, mangiferin concentration, and antioxidant activity.

Group	Group members	Characteristics	Average quantity of parameters of groups compared to the overall average values			
			TPC (131.2)	AOA (35.0)	MC (59.2)	EI (9.4)
Grade A	Koona, Kate	Above average total phenolic content, mangiferin concentration, antioxidant activity, and extraction index	153.9	20.7	99.2	9.8

Grade B	Kagoogwa, Takataka, Kawanda green, Kawanda wide	Above average total phenolic content and extraction index and below average mangiferin concentration and antioxidant activity	168.4	37.5	52.4	11.0

Grade C	Bire, Ngoogwe, Suu, Doodo red, Boribo, Sejjembe	Below average extraction index, mangiferin concentration, and total phenolic content but above average antioxidant activity	113.2	26.4	56.7	8.6

Grade D	Apple mango, MPI, Asante	Below average extraction index, total phenolic content, and mangiferin concentration and very low antioxidant activity	102.5	63.0	46.6	8.5

## Data Availability

The datasets used to support the findings of this study are included within the supplementary information file.

## Supplementary Materials

Supplementary Materials Table S1: relationship among the parameters. Table S2: Tukey's multiple comparisons of leaf TPC with source variety of *Mangifera indica*. Table S3: Tukey's multiple comparisons of leaf mangiferin concentration with the *Mangifera indica* variety. Table S4: validation of the HPLC method for quantification of mangiferin in *Mangifera indica* leaves. Table S5: characteristics of whole chromatogram fingerprints. Table S6: common peaks used to calculate fingerprints. Table S7: Tukey's multiple comparisons of leaf antioxidant activity with the *Mangifera indica* variety. Figure S1: interday repeatability of *Mangifera indica* leaf fingerprints (samples for Koona variety were used). Figure S2: intraday repeatability of *Mangifera indica* leaf fingerprints (samples for Koona variety were used).
