# Supplementary material for: Chemometric Classification of Mangifera indica L. Leaf Cultivars, Based on Selected Phytochemical Parameters; Implications for Standardization of the Pharmaceutical Raw Materials
Source: Evid Based Complement Alternat Med. 2023 Aug 4;2023:7245876. doi: 10.1155/2023/7245876 (PMC10421708; doi:10.1155/2023/7245876)
Supplement: Supplementary Materials — Table S1: relationship among the parameters. Table S2: Tukey's multiple comparisons of leaf TPC with source variety of Mangifera indica. Table S3: Tukey's multiple comparisons of leaf mangiferin concentration with the Mangifera indica variety. Table S4: validation of the HPLC method for quantification of mangiferin in Mangifera indica leaves. Table S5: characteristics of whole chromatogram fingerprints. Table S6: common peaks used to calculate fingerprints. Table S7: Tukey's multiple comparisons of leaf antioxidant activity with the Mangifera indica variety. Figure S1: interday repeatability of Mangifera indica leaf fingerprints (samples for Koona variety were used). Figure S2: intraday repeatability of Mangifera indica leaf fingerprints (samples for Koona variety were used). [file 7245876.f1.docx]

**Supplementary materials**

**Table S1: Relationship among the parameters**

| MF/TPC | TPC/AOA | TPC/EI | AOA/MC |
| --- | --- | --- | --- |
| 0.6 | 1.4 | 8.0 | 6,9 |
| 0.6 | 2.6 | 10.3 | 3,7 |
| 0.3 | 2.3 | 18.2 | 3,0 |
| 0.6 | 8.2 | 14.4 | 1,3 |
| 0.4 | 1.3 | 14.1 | 4,8 |
| 0.3 | 2.1 | 10.9 | 4,9 |
| 0.4 | 2.8 | 12.7 | 2,7 |
| 0.6 | 4.5 | 18.4 | 1,3 |
| 0.3 | 7.7 | 15.4 | 1,3 |
| 0.5 | 2.0 | 14.9 | 4,4 |
| 0.4 | 8.9 | 18.3 | 1,2 |
| 0.5 | 3.7 | 14.0 | 1,8 |
| 0.5 | 4.1 | 16.1 | 1,7 |
| 0.5 | 5.8 | 15.9 | 1,2 |
| 0.6 | 4.9 | 10.8 | 1,6 |
| Mean values 0.5 | 4.2 | 14.2 | 2,8 |

**Table S2: Tukey's multiple comparisons of leaf TPC with source variety of *Mangifera indica.***

| **Tukey's multiple comparisons test** | **Mean Diff.** | **95.00% CI of diff.** | **Significant?** | **Adjusted P Value** |
| --- | --- | --- | --- | --- |
| **Apple M vs. Sejjembe** | -10.02 | -19.94 to -0.09368 | Yes | 0.0468 |
| **Apple M vs. Kawanda green** | -13.63 | -23.55 to -3.704 | Yes | 0.0035 |
| **Apple M vs. Kate** | -35.54 | -45.46 to -25.62 | Yes | <0.0001 |
| **Apple M vs. Asante** | -2.380 | -12.30 to 7.541 | No | 0.9992 |
| **Apple M vs. Kawanda wide** | -12.56 | -22.48 to -2.639 | Yes | 0.0075 |
| **Apple M vs. Suu** | -19.37 | -29.29 to -9.449 | Yes | <0.0001 |
| **Apple M vs. Koona** | -38.66 | -48.58 to -28.74 | Yes | <0.0001 |
| **Apple M vs. Kagoogwa** | -21.34 | -31.26 to -11.42 | Yes | <0.0001 |
| **Apple M vs. MPI** | 9.360 | -0.5613 to 19.28 | No | 0.0740 |
| **Apple M vs. Takataka** | -13.79 | -23.71 to -3.869 | Yes | 0.0031 |
| **Apple M vs. Boribo** | -22.66 | -32.58 to -12.73 | Yes | <0.0001 |
| **Apple M vs. Ngoogwe** | 8.455 | -1.466 to 18.38 | No | 0.1362 |
| **Apple M vs. Bire** | -2.055 | -11.98 to 7.866 | No | 0.9998 |
| **Apple M vs. Doodo red** | -0.5750 | -10.50 to 9.346 | No | >0.9999 |
| **Sejjembe vs. Kawanda green** | -3.610 | -13.53 to 6.311 | No | 0.9662 |
| **Sejjembe vs. Kate** | -25.53 | -35.45 to -15.60 | Yes | <0.0001 |
| **Sejjembe vs. Asante** | 7.635 | -2.286 to 17.56 | No | 0.2282 |
| **Sejjembe vs. Kawanda wide** | -2.545 | -12.47 to 7.376 | No | 0.9984 |
| **Sejjembe vs. Suu** | -9.355 | -19.28 to 0.5663 | No | 0.0743 |
| **Sejjembe vs. Koona** | -28.65 | -38.57 to -18.72 | Yes | <0.0001 |
| **Sejjembe vs. Kagoogwa** | -11.33 | -21.25 to -1.404 | Yes | 0.0183 |
| **Sejjembe vs. MPI** | 19.38 | 9.454 to 29.30 | Yes | <0.0001 |
| **Sejjembe vs. Takataka** | -3.775 | -13.70 to 6.146 | No | 0.9534 |
| **Sejjembe vs. Boribo** | -12.64 | -22.56 to -2.719 | Yes | 0.0071 |
| **Sejjembe vs. Ngoogwe** | 18.47 | 8.549 to 28.39 | Yes | 0.0001 |
| **Sejjembe vs. Bire** | 7.960 | -1.961 to 17.88 | No | 0.1869 |
| **Sejjembe vs. Doodo red** | 9.440 | -0.4813 to 19.36 | No | 0.0700 |
| **Kawanda green vs. Kate** | -21.92 | -31.84 to -11.99 | Yes | <0.0001 |
| **Kawanda green vs. Asante** | 11.25 | 1.324 to 21.17 | Yes | 0.0194 |
| **Kawanda green vs. Kawanda wide** | 1.065 | -8.856 to 10.99 | No | >0.9999 |
| **Kawanda green vs. Suu** | -5.745 | -15.67 to 4.176 | No | 0.5965 |
| **Kawanda green vs. Koona** | -25.04 | -34.96 to -15.11 | Yes | <0.0001 |
| **Kawanda green vs. Kagoogwa** | -7.715 | -17.64 to 2.206 | No | 0.2174 |
| **Kawanda green vs. MPI** | 22.99 | 13.06 to 32.91 | Yes | <0.0001 |
| **Kawanda green vs. Takataka** | -0.1650 | -10.09 to 9.756 | No | >0.9999 |
| **Kawanda green vs. Boribo** | -9.030 | -18.95 to 0.8913 | No | 0.0928 |
| **Kawanda green vs. Ngoogwe** | 22.08 | 12.16 to 32.00 | Yes | <0.0001 |
| **Kawanda green vs. Bire** | 11.57 | 1.649 to 21.49 | Yes | 0.0154 |
| **Kawanda green vs. Doodo red** | 13.05 | 3.129 to 22.97 | Yes | 0.0053 |
| **Kate vs. Asante** | 33.16 | 23.24 to 43.08 | Yes | <0.0001 |
| **Kate vs. Kawanda wide** | 22.98 | 13.06 to 32.90 | Yes | <0.0001 |
| **Kate vs. Suu** | 16.17 | 6.249 to 26.09 | Yes | 0.0006 |
| **Kate vs. Koona** | -3.120 | -13.04 to 6.801 | No | 0.9894 |
| **Kate vs. Kagoogwa** | 14.20 | 4.279 to 24.12 | Yes | 0.0023 |
| **Kate vs. MPI** | 44.90 | 34.98 to 54.82 | Yes | <0.0001 |
| **Kate vs. Takataka** | 21.75 | 11.83 to 31.67 | Yes | <0.0001 |
| **Kate vs. Boribo** | 12.89 | 2.964 to 22.81 | Yes | 0.0060 |
| **Kate vs. Ngoogwe** | 44.00 | 34.07 to 53.92 | Yes | <0.0001 |
| **Kate vs. Bire** | 33.49 | 23.56 to 43.41 | Yes | <0.0001 |
| **Kate vs. Doodo red** | 34.97 | 25.04 to 44.89 | Yes | <0.0001 |
| **Asante vs. Kawanda wide** | -10.18 | -20.10 to -0.2587 | Yes | 0.0416 |
| **Asante vs. Suu** | -16.99 | -26.91 to -7.069 | Yes | 0.0004 |
| **Asante vs. Koona** | -36.28 | -46.20 to -26.36 | Yes | <0.0001 |
| **Asante vs. Kagoogwa** | -18.96 | -28.88 to -9.039 | Yes | <0.0001 |
| **Asante vs. MPI** | 11.74 | 1.819 to 21.66 | Yes | 0.0136 |
| **Asante vs. Takataka** | -11.41 | -21.33 to -1.489 | Yes | 0.0172 |
| **Asante vs. Boribo** | -20.28 | -30.20 to -10.35 | Yes | <0.0001 |
| **Asante vs. Ngoogwe** | 10.84 | 0.9137 to 20.76 | Yes | 0.0261 |
| **Asante vs. Bire** | 0.3250 | -9.596 to 10.25 | No | >0.9999 |
| **Asante vs. Doodo red** | 1.805 | -8.116 to 11.73 | No | >0.9999 |
| **Kawanda wide vs. Suu** | -6.810 | -16.73 to 3.111 | No | 0.3641 |
| **Kawanda wide vs. Koona** | -26.10 | -36.02 to -16.18 | Yes | <0.0001 |
| **Kawanda wide vs. Kagoogwa** | -8.780 | -18.70 to 1.141 | No | 0.1098 |
| **Kawanda wide vs. MPI** | 21.92 | 12.00 to 31.84 | Yes | <0.0001 |
| **Kawanda wide vs. Takataka** | -1.230 | -11.15 to 8.691 | No | >0.9999 |
| **Kawanda wide vs. Boribo** | -10.10 | -20.02 to -0.1737 | Yes | 0.0442 |
| **Kawanda wide vs. Ngoogwe** | 21.02 | 11.09 to 30.94 | Yes | <0.0001 |
| **Kawanda wide vs. Bire** | 10.51 | 0.5837 to 20.43 | Yes | 0.0330 |
| **Kawanda wide vs. Doodo red** | 11.99 | 2.064 to 21.91 | Yes | 0.0114 |
| **Suu vs. Koona** | -19.29 | -29.21 to -9.369 | Yes | <0.0001 |
| **Suu vs. Kagoogwa** | -1.970 | -11.89 to 7.951 | No | 0.9999 |
| **Suu vs. MPI** | 28.73 | 18.81 to 38.65 | Yes | <0.0001 |
| **Suu vs. Takataka** | 5.580 | -4.341 to 15.50 | No | 0.6351 |
| **Suu vs. Boribo** | -3.285 | -13.21 to 6.636 | No | 0.9837 |
| **Suu vs. Ngoogwe** | 27.83 | 17.90 to 37.75 | Yes | <0.0001 |
| **Suu vs. Bire** | 17.32 | 7.394 to 27.24 | Yes | 0.0003 |
| **Suu vs. Doodo red** | 18.80 | 8.874 to 28.72 | Yes | 0.0001 |
| **Koona vs. Kagoogwa** | 17.32 | 7.399 to 27.24 | Yes | 0.0003 |
| **Koona vs. MPI** | 48.02 | 38.10 to 57.94 | Yes | <0.0001 |
| **Koona vs. Takataka** | 24.87 | 14.95 to 34.79 | Yes | <0.0001 |
| **Koona vs. Boribo** | 16.01 | 6.084 to 25.93 | Yes | 0.0007 |
| **Koona vs. Ngoogwe** | 47.12 | 37.19 to 57.04 | Yes | <0.0001 |
| **Koona vs. Bire** | 36.61 | 26.68 to 46.53 | Yes | <0.0001 |
| **Koona vs. Doodo red** | 38.09 | 28.16 to 48.01 | Yes | <0.0001 |
| **Kagoogwa vs. MPI** | 30.70 | 20.78 to 40.62 | Yes | <0.0001 |
| **Kagoogwa vs. Takataka** | 7.550 | -2.371 to 17.47 | No | 0.2401 |
| **Kagoogwa vs. Boribo** | -1.315 | -11.24 to 8.606 | No | >0.9999 |
| **Kagoogwa vs. Ngoogwe** | 29.80 | 19.87 to 39.72 | Yes | <0.0001 |
| **Kagoogwa vs. Bire** | 19.29 | 9.364 to 29.21 | Yes | <0.0001 |
| **Kagoogwa vs. Doodo red** | 20.77 | 10.84 to 30.69 | Yes | <0.0001 |
| **MPI vs. Takataka** | -23.15 | -33.07 to -13.23 | Yes | <0.0001 |
| **MPI vs. Boribo** | -32.02 | -41.94 to -22.09 | Yes | <0.0001 |
| **MPI vs. Ngoogwe** | -0.9050 | -10.83 to 9.016 | No | >0.9999 |
| **MPI vs. Bire** | -11.42 | -21.34 to -1.494 | Yes | 0.0172 |
| **MPI vs. Doodo red** | -9.935 | -19.86 to -0.01368 | Yes | 0.0495 |
| **Takataka vs. Boribo** | -8.865 | -18.79 to 1.056 | No | 0.1037 |
| **Takataka vs. Ngoogwe** | 22.25 | 12.32 to 32.17 | Yes | <0.0001 |
| **Takataka vs. Bire** | 11.74 | 1.814 to 21.66 | Yes | 0.0136 |
| **Takataka vs. Doodo red** | 13.22 | 3.294 to 23.14 | Yes | 0.0047 |
| **Boribo vs. Ngoogwe** | 31.11 | 21.19 to 41.03 | Yes | <0.0001 |
| **Boribo vs. Bire** | 20.60 | 10.68 to 30.52 | Yes | <0.0001 |
| **Boribo vs. Doodo red** | 22.08 | 12.16 to 32.00 | Yes | <0.0001 |
| **Ngoogwe vs. Bire** | -10.51 | -20.43 to -0.5887 | Yes | 0.0329 |
| **Ngoogwe vs. Doodo red** | -9.030 | -18.95 to 0.8913 | No | 0.0928 |
| **Bire vs. Doodo red** | 1.480 | -8.441 to 11.40 | No | >0.9999 |

**Table S3:** Tukey's multiple comparisons of leaf Mangiferin concentration with the *Mangifera indica* variety.

| Tukey's multiple comparisons test | Mean Diff. | 95.00% CI of diff. | Below threshold? | Adjusted P Value |
| --- | --- | --- | --- | --- |
| Apple M vs. Sejjembe | -9.870 | -17.81 to -1.929 | Yes | 0.0089 |
| Apple M vs. Kawanda green | -13.61 | -21.55 to -5.669 | Yes | 0.0003 |
| Apple M vs. Kate | -35.15 | -43.09 to -27.21 | Yes | <0.0001 |
| Apple M vs. Asante | -2.270 | -10.21 to 5.671 | No | 0.9954 |
| Apple M vs. Kawanda wide | -12.36 | -20.30 to -4.419 | Yes | 0.0010 |
| Apple M vs. Suu | -20.63 | -28.57 to -12.69 | Yes | <0.0001 |
| Apple M vs. Koona | -38.30 | -46.24 to -30.36 | Yes | <0.0001 |
| Apple M vs. Kagoogwa | -21.08 | -29.02 to -13.14 | Yes | <0.0001 |
| Apple M vs. MPI | 8.970 | 1.029 to 16.91 | Yes | 0.0199 |
| Apple M vs. Takataka | -13.72 | -21.66 to -5.779 | Yes | 0.0003 |
| Apple M vs. Boribo | -22.38 | -30.32 to -14.44 | Yes | <0.0001 |
| Apple M vs. Ngoogwe | 8.020 | 0.07920 to 15.96 | Yes | 0.0466 |
| Apple M vs. Bire | 0.01000 | -7.931 to 7.951 | No | >0.9999 |
| Apple M vs. Doodo red | -0.5200 | -8.461 to 7.421 | No | >0.9999 |
| Sejjembe vs. Kawanda green | -3.740 | -11.68 to 4.201 | No | 0.8308 |
| Sejjembe vs. Kate | -25.28 | -33.22 to -17.34 | Yes | <0.0001 |
| Sejjembe vs. Asante | 7.600 | -0.3408 to 15.54 | No | 0.0674 |
| Sejjembe vs. Kawanda wide | -2.490 | -10.43 to 5.451 | No | 0.9896 |
| Sejjembe vs. Suu | -10.76 | -18.70 to -2.819 | Yes | 0.0040 |
| Sejjembe vs. Koona | -28.43 | -36.37 to -20.49 | Yes | <0.0001 |
| Sejjembe vs. Kagoogwa | -11.21 | -19.15 to -3.269 | Yes | 0.0027 |
| Sejjembe vs. MPI | 18.84 | 10.90 to 26.78 | Yes | <0.0001 |
| Sejjembe vs. Takataka | -3.850 | -11.79 to 4.091 | No | 0.8045 |
| Sejjembe vs. Boribo | -12.51 | -20.45 to -4.569 | Yes | 0.0009 |
| Sejjembe vs. Ngoogwe | 17.89 | 9.949 to 25.83 | Yes | <0.0001 |
| Sejjembe vs. Bire | 9.880 | 1.939 to 17.82 | Yes | 0.0088 |
| Sejjembe vs. Doodo red | 9.350 | 1.409 to 17.29 | Yes | 0.0142 |
| Kawanda green vs. Kate | -21.54 | -29.48 to -13.60 | Yes | <0.0001 |
| Kawanda green vs. Asante | 11.34 | 3.399 to 19.28 | Yes | 0.0024 |
| Kawanda green vs. Kawanda wide | 1.250 | -6.691 to 9.191 | No | >0.9999 |
| Kawanda green vs. Suu | -7.020 | -14.96 to 0.9208 | No | 0.1105 |
| Kawanda green vs. Koona | -24.69 | -32.63 to -16.75 | Yes | <0.0001 |
| Kawanda green vs. Kagoogwa | -7.470 | -15.41 to 0.4708 | No | 0.0754 |
| Kawanda green vs. MPI | 22.58 | 14.64 to 30.52 | Yes | <0.0001 |
| Kawanda green vs. Takataka | -0.1100 | -8.051 to 7.831 | No | >0.9999 |
| Kawanda green vs. Boribo | -8.770 | -16.71 to -0.8292 | Yes | 0.0239 |
| Kawanda green vs. Ngoogwe | 21.63 | 13.69 to 29.57 | Yes | <0.0001 |
| Kawanda green vs. Bire | 13.62 | 5.679 to 21.56 | Yes | 0.0003 |
| Kawanda green vs. Doodo red | 13.09 | 5.149 to 21.03 | Yes | 0.0005 |
| Kate vs. Asante | 32.88 | 24.94 to 40.82 | Yes | <0.0001 |
| Kate vs. Kawanda wide | 22.79 | 14.85 to 30.73 | Yes | <0.0001 |
| Kate vs. Suu | 14.52 | 6.579 to 22.46 | Yes | 0.0002 |
| Kate vs. Koona | -3.150 | -11.09 to 4.791 | No | 0.9382 |
| Kate vs. Kagoogwa | 14.07 | 6.129 to 22.01 | Yes | 0.0002 |
| Kate vs. MPI | 44.12 | 36.18 to 52.06 | Yes | <0.0001 |
| Kate vs. Takataka | 21.43 | 13.49 to 29.37 | Yes | <0.0001 |
| Kate vs. Boribo | 12.77 | 4.829 to 20.71 | Yes | 0.0007 |
| Kate vs. Ngoogwe | 43.17 | 35.23 to 51.11 | Yes | <0.0001 |
| Kate vs. Bire | 35.16 | 27.22 to 43.10 | Yes | <0.0001 |
| Kate vs. Doodo red | 34.63 | 26.69 to 42.57 | Yes | <0.0001 |
| Asante vs. Kawanda wide | -10.09 | -18.03 to -2.149 | Yes | 0.0073 |
| Asante vs. Suu | -18.36 | -26.30 to -10.42 | Yes | <0.0001 |
| Asante vs. Koona | -36.03 | -43.97 to -28.09 | Yes | <0.0001 |
| Asante vs. Kagoogwa | -18.81 | -26.75 to -10.87 | Yes | <0.0001 |
| Asante vs. MPI | 11.24 | 3.299 to 19.18 | Yes | 0.0026 |
| Asante vs. Takataka | -11.45 | -19.39 to -3.509 | Yes | 0.0022 |
| Asante vs. Boribo | -20.11 | -28.05 to -12.17 | Yes | <0.0001 |
| Asante vs. Ngoogwe | 10.29 | 2.349 to 18.23 | Yes | 0.0061 |
| Asante vs. Bire | 2.280 | -5.661 to 10.22 | No | 0.9952 |
| Asante vs. Doodo red | 1.750 | -6.191 to 9.691 | No | 0.9997 |
| Kawanda wide vs. Suu | -8.270 | -16.21 to -0.3292 | Yes | 0.0374 |
| Kawanda wide vs. Koona | -25.94 | -33.88 to -18.00 | Yes | <0.0001 |
| Kawanda wide vs. Kagoogwa | -8.720 | -16.66 to -0.7792 | Yes | 0.0250 |
| Kawanda wide vs. MPI | 21.33 | 13.39 to 29.27 | Yes | <0.0001 |
| Kawanda wide vs. Takataka | -1.360 | -9.301 to 6.581 | No | >0.9999 |
| Kawanda wide vs. Boribo | -10.02 | -17.96 to -2.079 | Yes | 0.0077 |
| Kawanda wide vs. Ngoogwe | 20.38 | 12.44 to 28.32 | Yes | <0.0001 |
| Kawanda wide vs. Bire | 12.37 | 4.429 to 20.31 | Yes | 0.0010 |
| Kawanda wide vs. Doodo red | 11.84 | 3.899 to 19.78 | Yes | 0.0015 |
| Suu vs. Koona | -17.67 | -25.61 to -9.729 | Yes | <0.0001 |
| Suu vs. Kagoogwa | -0.4500 | -8.391 to 7.491 | No | >0.9999 |
| Suu vs. MPI | 29.60 | 21.66 to 37.54 | Yes | <0.0001 |
| Suu vs. Takataka | 6.910 | -1.031 to 14.85 | No | 0.1211 |
| Suu vs. Boribo | -1.750 | -9.691 to 6.191 | No | 0.9997 |
| Suu vs. Ngoogwe | 28.65 | 20.71 to 36.59 | Yes | <0.0001 |
| Suu vs. Bire | 20.64 | 12.70 to 28.58 | Yes | <0.0001 |
| Suu vs. Doodo red | 20.11 | 12.17 to 28.05 | Yes | <0.0001 |
| Koona vs. Kagoogwa | 17.22 | 9.279 to 25.16 | Yes | <0.0001 |
| Koona vs. MPI | 47.27 | 39.33 to 55.21 | Yes | <0.0001 |
| Koona vs. Takataka | 24.58 | 16.64 to 32.52 | Yes | <0.0001 |
| Koona vs. Boribo | 15.92 | 7.979 to 23.86 | Yes | <0.0001 |
| Koona vs. Ngoogwe | 46.32 | 38.38 to 54.26 | Yes | <0.0001 |
| Koona vs. Bire | 38.31 | 30.37 to 46.25 | Yes | <0.0001 |
| Koona vs. Doodo red | 37.78 | 29.84 to 45.72 | Yes | <0.0001 |
| Kagoogwa vs. MPI | 30.05 | 22.11 to 37.99 | Yes | <0.0001 |
| Kagoogwa vs. Takataka | 7.360 | -0.5808 to 15.30 | No | 0.0829 |
| Kagoogwa vs. Boribo | -1.300 | -9.241 to 6.641 | No | >0.9999 |
| Kagoogwa vs. Ngoogwe | 29.10 | 21.16 to 37.04 | Yes | <0.0001 |
| Kagoogwa vs. Bire | 21.09 | 13.15 to 29.03 | Yes | <0.0001 |
| Kagoogwa vs. Doodo red | 20.56 | 12.62 to 28.50 | Yes | <0.0001 |
| MPI vs. Takataka | -22.69 | -30.63 to -14.75 | Yes | <0.0001 |
| MPI vs. Boribo | -31.35 | -39.29 to -23.41 | Yes | <0.0001 |
| MPI vs. Ngoogwe | -0.9500 | -8.891 to 6.991 | No | >0.9999 |
| MPI vs. Bire | -8.960 | -16.90 to -1.019 | Yes | 0.0201 |
| MPI vs. Doodo red | -9.490 | -17.43 to -1.549 | Yes | 0.0125 |
| Takataka vs. Boribo | -8.660 | -16.60 to -0.7192 | Yes | 0.0264 |
| Takataka vs. Ngoogwe | 21.74 | 13.80 to 29.68 | Yes | <0.0001 |
| Takataka vs. Bire | 13.73 | 5.789 to 21.67 | Yes | 0.0003 |
| Takataka vs. Doodo red | 13.20 | 5.259 to 21.14 | Yes | 0.0005 |
| Boribo vs. Ngoogwe | 30.40 | 22.46 to 38.34 | Yes | <0.0001 |
| Boribo vs. Bire | 22.39 | 14.45 to 30.33 | Yes | <0.0001 |
| Boribo vs. Doodo red | 21.86 | 13.92 to 29.80 | Yes | <0.0001 |
| Ngoogwe vs. Bire | -8.010 | -15.95 to -0.06920 | Yes | 0.0470 |
| Ngoogwe vs. Doodo red | -8.540 | -16.48 to -0.5992 | Yes | 0.0293 |
| Bire vs. Doodo red | -0.5300 | -8.471 to 7.411 | No | >0.9999 |
|  |  |  |  |  |

**Table S4: Validation of the HPLC method for quantification of mangiferin in *Mangifera indica* leaves**

| Method validation parameters for the HPLC method | n | Value of parameter | Reference values (AOAC) |
| --- | --- | --- | --- |
| **Recovery (%)** | 9 | 99.4±4.16 | 92–105 |
| **Repeatability (RSD %)** |  |  |  |
| Intraday | 12 | 0.16±0.04 | 1.5-2 |
| Inter-day | 12 | 0.32±0.22 | 3-4 |
| **Linearity** |  |  |  |
| Regression equation |  | Y=18709.81X-99470.48 |  |
| R^2^ |  | 0.998 |  |
| **Sensitivity** |  |  |  |
| **LOD (µg/mL)** |  | 7.36 |  |
| **LOQ (µg/mL)** |  | 22.3 |  |

**Table S5:** Characteristics of whole chromatogram fingerprints

| Variety | Apple M | Asante | Bire | Boribo | Doodo red | Takataka | Kagoogwa | Kate | Kawanda green | Kawanda wide | Koona | MPI | Ngoogwe | Sejjembe | Suu | Averages | SD |
| --- | --- | --- | --- | --- | --- | --- | --- | --- | --- | --- | --- | --- | --- | --- | --- | --- | --- |
| No of peaks | 25 | 20 | 23 | 24 | 25 | 25 | 20 | 23 | 19 | 29 | 25 | 28 | 23 | 22 | 21 | 23.5 | 2.9 |
| Total area | 1948744 | 1568633 | 2478665 | 1937371 | 2355795 | 2833600 | 2193084 | 5863448 | 2678994 | 2301693 | 3043927 | 2000401 | 1811403 | 1883593 | 1962421 | 2457451 | 1026790 |

**Table S6: Common peaks used to calculate fingerprints**

| Peak number | 1 | 2 | 3 | 4 | 5 | 6 | 7 | 8 | 9 | 10 |  |
| --- | --- | --- | --- | --- | --- | --- | --- | --- | --- | --- | --- |
| Retention time | 2.607 | 2.799 | 3.007 | 3.595 | 4.195 | 4.565 | 5.489 | 6.021 | 6.512 | 15.828 | Total area |
| Peak areas | | | | | | | | | | | |
| Kawanda wide (reference) | 6724 | 5855 | 48257 | 17665 | 51060 | 31838 | 20775 | 268237 | 19451 | 787799 | 1257661 |
| Apple mango | 11323 | 11040 | 39909 | 16507 | 35181 | 14750 | 14717 | 144109 | 20125 | 966630 | 1274291 |
| Sejjembe | 29622 | 14466 | 88357 | 22785 | 61338 | 61338 | 16834 | 36615 | 25146 | 996217 | 1352718 |
| Kawanda green | 5500 | 6295 | 28006 | 12679 | 41926 | 20702 | 62572 | 263930 | 28235 | 1575647 | 2045492 |
| Kate | 14941 | 5579 | 86596 | 90976 | 150532 | 48181 | 57651 | 200670 | 56134 | 2999536 | 3710796 |
| Asante | 1883 | 2978 | 34802 | 6106 | 37878 | 13250 | 11917 | 137923 | 15641 | 699076 | 961454 |
| Suu | 13580 | 7709 | 51652 | 24357 | 102538 | 28808 | 28808 | 141148 | 19332 | 795323 | 1213255 |
| Koona | 64858 | 13146 | 72174 | 25001 | 123771 | 63799 | 51950 | 46229 | 48480 | 1684417 | 2193825 |
| Kagoogwa | 30072 | 8664 | 53353 | 23317 | 56311 | 17056 | 29099 | 245416 | 12952 | 955005 | 1431245 |
| MPI | 53206 | 16293 | 71632 | 16998 | 67737 | 39567 | 40880 | 96508 | 24293 | 1039838 | 1466952 |
| Takataka | 12388 | 9393 | 52808 | 54529 | 81609 | 21984 | 32062 | 79492 | 39765 | 1314151 | 1698181 |
| Boribo | 11080 | 6295 | 45009 | 29931 | 59765 | 55201 | 34609 | 58715 | 21996 | 1034962 | 1357563 |
| Ngoogwe | 16089 | 10816 | 38678 | 13343 | 57562 | 46335 | 33694 | 44917 | 23486 | 1159149 | 1444069 |
| Bire | 23852 | 15584 | 50710 | 33913 | 63670 | 25552 | 35720 | 132430 | 44361 | 1318352 | 1744144 |
| Doodo red | 9921 | 8982 | 56657 | 48822 | 106002 | 88008 | 43946 | 70803 | 29476 | 1168948 | 1631565 |
| Mean | 20335.93 | 9539.667 | 54573.33 | 29128.6 | 73125.33 | 38424.6 | 34348.93 | 131142.8 | 28591.53 | 1233003 | 1652214 |
| STD | 17795.01 | 3974.215 | 17920.02 | 21519.35 | 33464.23 | 21720.18 | 15188.3 | 80609.42 | 12766.5 | 561943.8 | 652910.2 |
| (%) of total peaks area | 1.2 | 0.6 | 3.3 | 1.8 | 4.4 | 2.3 | 2.1 | 7.9 | 1.7 | 74.6 |  |

**
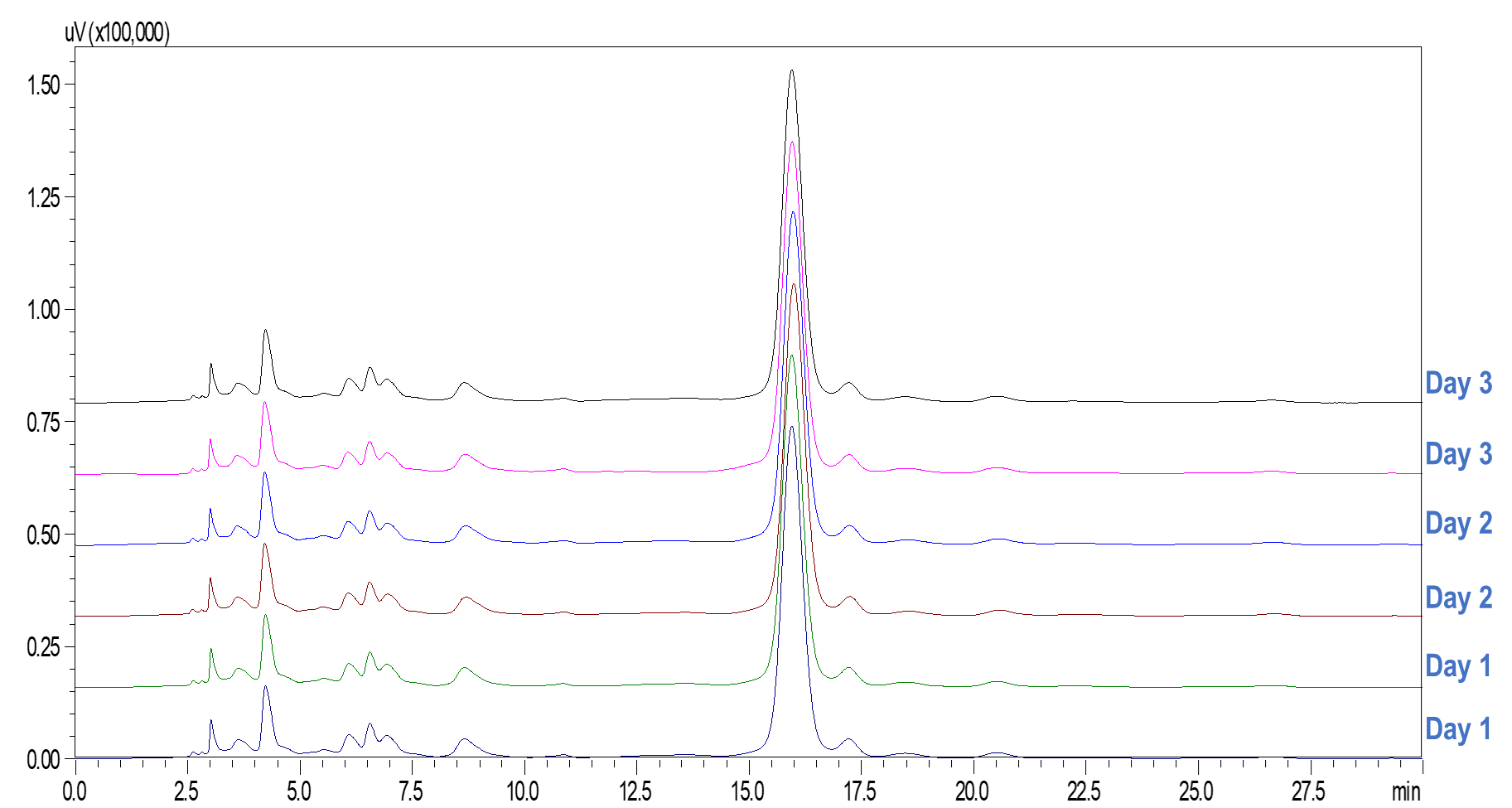
**

**Figure S1:** Inter day repeatability of *Mangifera indica* leaf fingerprints, samples for Koona variety were used.

**
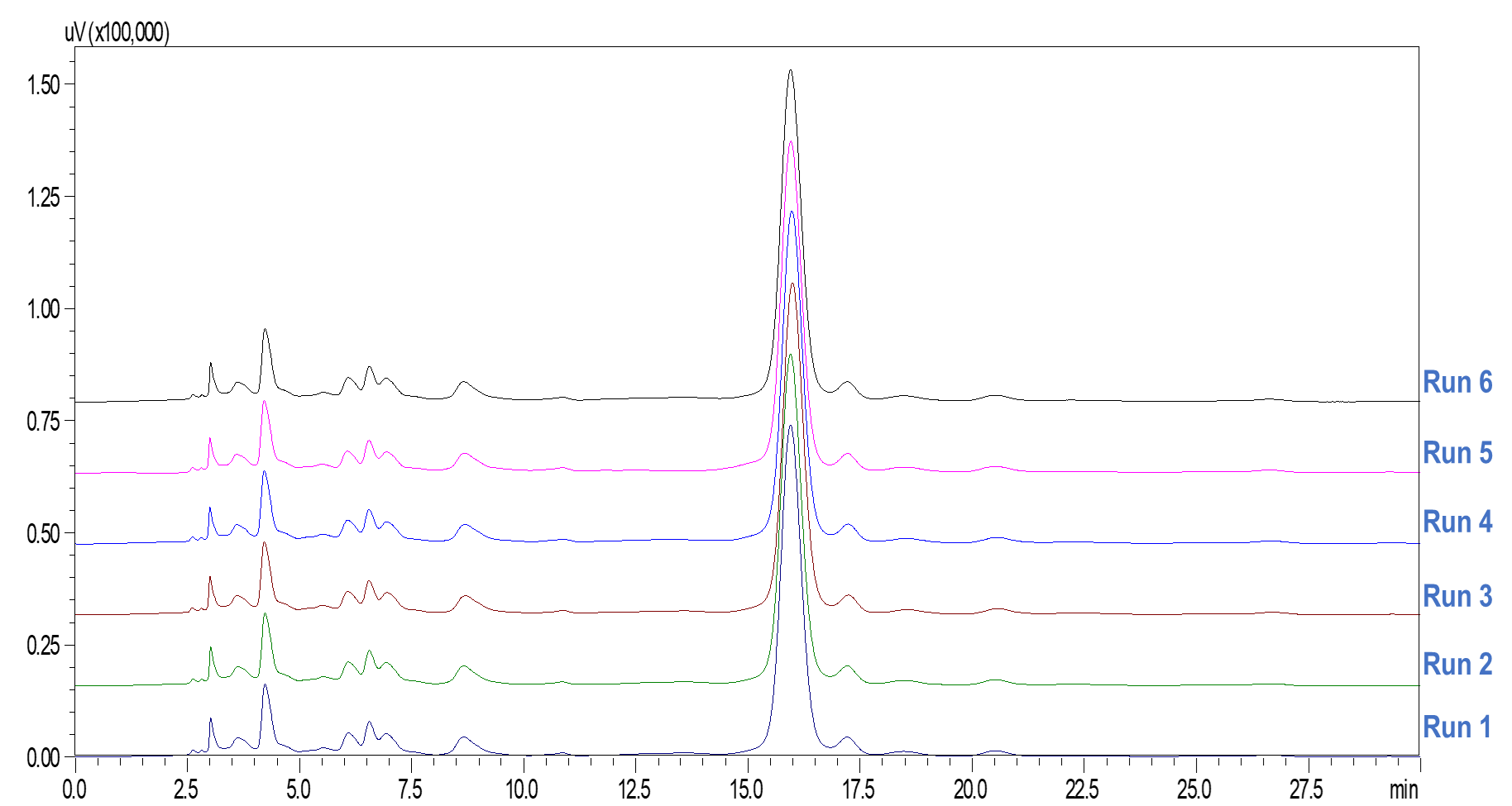
**

**Figure S2:** Intraday repeatability of *Mangifera indica* leaf fingerprints, samples for Koona variety were used.

**Table S7: Tukey's multiple comparisons of leaf Antioxidant activity with source variety of *Mangifera indica.***

| Number of families | 1 |  |  |
| --- | --- | --- | --- |
| Number of comparisons per family | 105 |  |  |
| Alpha | 0,05 |  |  |
|  |  |  |  |
| Tukey's multiple comparisons test | Mean Diff, | 95,00% CI of diff, | Adjusted P Value |
| Apple M vs. Sejjembe | 17,44 | -12,11 to 46,99 | 0,6787 |
| Apple M vs. Kawanda green | 1,747 | -27,80 to 31,30 | >0,9999 |
| Apple M vs. Kate | 37,06 | 7,508 to 66,61 | 0,0049 |
| Apple M vs. Asante | -12,26 | -41,81 to 17,29 | 0,9613 |
| Apple M vs. Kawanda wide | 1,610 | -27,94 to 31,16 | >0,9999 |
| Apple M vs. Suu | 21,72 | -7,835 to 51,27 | 0,3467 |
| Apple M vs. Koona | 32,33 | 2,775 to 61,88 | 0,0219 |
| Apple M vs. Kagoogwa | 35,67 | 6,118 to 65,22 | 0,0076 |
| Apple M vs. MPI | -10,47 | -40,02 to 19,08 | 0,9896 |
| Apple M vs. Takataka | 32,59 | 3,035 to 62,14 | 0,0202 |
| Apple M vs. Boribo | 30,24 | 0,6885 to 59,79 | 0,0409 |
| Apple M vs. Ngoogwe | 28,48 | -1,075 to 58,03 | 0,0678 |
| Apple M vs. Bire | 36,70 | 7,145 to 66,25 | 0,0055 |
| Apple M vs. Doodo red | 38,60 | 9,045 to 68,15 | 0,0029 |
| Sejjembe vs. Kawanda green | -15,69 | -45,24 to 13,86 | 0,8065 |
| Sejjembe vs. Kate | 19,62 | -9,932 to 49,17 | 0,5028 |
| Sejjembe vs. Asante | -29,70 | -59,25 to -0,1485 | 0,0479 |
| Sejjembe vs. Kawanda wide | -15,83 | -45,38 to 13,72 | 0,7974 |
| Sejjembe vs. Suu | 4,277 | -25,27 to 33,83 | >0,9999 |
| Sejjembe vs. Koona | 14,89 | -14,66 to 44,44 | 0,8559 |
| Sejjembe vs. Kagoogwa | 18,23 | -11,32 to 47,78 | 0,6154 |
| Sejjembe vs. MPI | -27,91 | -57,46 to 1,642 | 0,0793 |
| Sejjembe vs. Takataka | 15,15 | -14,40 to 44,70 | 0,8408 |
| Sejjembe vs. Boribo | 12,80 | -16,75 to 42,35 | 0,9466 |
| Sejjembe vs. Ngoogwe | 11,04 | -18,51 to 40,59 | 0,9835 |
| Sejjembe vs. Bire | 19,26 | -10,29 to 48,81 | 0,5320 |
| Sejjembe vs. Doodo red | 21,16 | -8,395 to 50,71 | 0,3857 |
| Kawanda green vs. Kate | 35,31 | 5,762 to 64,86 | 0,0086 |
| Kawanda green vs. Asante | -14,01 | -43,56 to 15,54 | 0,9009 |
| Kawanda green vs. Kawanda wide | -0,1367 | -29,69 to 29,41 | >0,9999 |
| Kawanda green vs. Suu | 19,97 | -9,582 to 49,52 | 0,4751 |
| Kawanda green vs. Koona | 30,58 | 1,028 to 60,13 | 0,0370 |
| Kawanda green vs. Kagoogwa | 33,92 | 4,372 to 63,47 | 0,0133 |
| Kawanda green vs. MPI | -12,22 | -41,77 to 17,33 | 0,9623 |
| Kawanda green vs. Takataka | 30,84 | 1,288 to 60,39 | 0,0343 |
| Kawanda green vs. Boribo | 28,49 | -1,058 to 58,04 | 0,0675 |
| Kawanda green vs. Ngoogwe | 26,73 | -2,822 to 56,28 | 0,1088 |
| Kawanda green vs. Bire | 34,95 | 5,398 to 64,50 | 0,0096 |
| Kawanda green vs. Doodo red | 36,85 | 7,298 to 66,40 | 0,0052 |
| Kate vs. Asante | -49,32 | -78,87 to -19,77 | <0,0001 |
| Kate vs. Kawanda wide | -35,45 | -65,00 to -5,898 | 0,0082 |
| Kate vs. Suu | -15,34 | -44,89 to 14,21 | 0,8288 |
| Kate vs. Koona | -4,733 | -34,28 to 24,82 | >0,9999 |
| Kate vs. Kagoogwa | -1,390 | -30,94 to 28,16 | >0,9999 |
| Kate vs. MPI | -47,53 | -77,08 to -17,98 | 0,0001 |
| Kate vs. Takataka | -4,473 | -34,02 to 25,08 | >0,9999 |
| Kate vs. Boribo | -6,820 | -36,37 to 22,73 | 0,9999 |
| Kate vs. Ngoogwe | -8,583 | -38,13 to 20,97 | 0,9985 |
| Kate vs. Bire | -0,3633 | -29,91 to 29,19 | >0,9999 |
| Kate vs. Doodo red | 1,537 | -28,01 to 31,09 | >0,9999 |
| Asante vs. Kawanda wide | 13,87 | -15,68 to 43,42 | 0,9070 |
| Asante vs. Suu | 33,98 | 4,425 to 63,53 | 0,0131 |
| Asante vs. Koona | 44,59 | 15,04 to 74,14 | 0,0004 |
| Asante vs. Kagoogwa | 47,93 | 18,38 to 77,48 | 0,0001 |
| Asante vs. MPI | 1,790 | -27,76 to 31,34 | >0,9999 |
| Asante vs. Takataka | 44,85 | 15,30 to 74,40 | 0,0004 |
| Asante vs. Boribo | 42,50 | 12,95 to 72,05 | 0,0008 |
| Asante vs. Ngoogwe | 40,74 | 11,19 to 70,29 | 0,0014 |
| Asante vs. Bire | 48,96 | 19,41 to 78,51 | <0,0001 |
| Asante vs. Doodo red | 50,86 | 21,31 to 80,41 | <0,0001 |
| Kawanda wide vs. Suu | 20,11 | -9,445 to 49,66 | 0,4644 |
| Kawanda wide vs. Koona | 30,72 | 1,165 to 60,27 | 0,0356 |
| Kawanda wide vs. Kagoogwa | 34,06 | 4,508 to 63,61 | 0,0128 |
| Kawanda wide vs. MPI | -12,08 | -41,63 to 17,47 | 0,9655 |
| Kawanda wide vs. Takataka | 30,98 | 1,425 to 60,53 | 0,0329 |
| Kawanda wide vs. Boribo | 28,63 | -0,9215 to 58,18 | 0,0650 |
| Kawanda wide vs. Ngoogwe | 26,87 | -2,685 to 56,42 | 0,1050 |
| Kawanda wide vs. Bire | 35,09 | 5,535 to 64,64 | 0,0092 |
| Kawanda wide vs. Doodo red | 36,99 | 7,435 to 66,54 | 0,0050 |
| Suu vs. Koona | 10,61 | -18,94 to 40,16 | 0,9883 |
| Suu vs. Kagoogwa | 13,95 | -15,60 to 43,50 | 0,9033 |
| Suu vs. MPI | -32,19 | -61,74 to -2,635 | 0,0228 |
| Suu vs. Takataka | 10,87 | -18,68 to 40,42 | 0,9855 |
| Suu vs. Boribo | 8,523 | -21,03 to 38,07 | 0,9986 |
| Suu vs. Ngoogwe | 6,760 | -22,79 to 36,31 | 0,9999 |
| Suu vs. Bire | 14,98 | -14,57 to 44,53 | 0,8506 |
| Suu vs. Doodo red | 16,88 | -12,67 to 46,43 | 0,7221 |
| Koona vs. Kagoogwa | 3,343 | -26,21 to 32,89 | >0,9999 |
| Koona vs. MPI | -42,80 | -72,35 to -13,25 | 0,0007 |
| Koona vs. Takataka | 0,2600 | -29,29 to 29,81 | >0,9999 |
| Koona vs. Boribo | -2,087 | -31,64 to 27,46 | >0,9999 |
| Koona vs. Ngoogwe | -3,850 | -33,40 to 25,70 | >0,9999 |
| Koona vs. Bire | 4,370 | -25,18 to 33,92 | >0,9999 |
| Koona vs. Doodo red | 6,270 | -23,28 to 35,82 | >0,9999 |
| Kagoogwa vs. MPI | -46,14 | -75,69 to -16,59 | 0,0002 |
| Kagoogwa vs. Takataka | -3,083 | -32,63 to 26,47 | >0,9999 |
| Kagoogwa vs. Boribo | -5,430 | -34,98 to 24,12 | >0,9999 |
| Kagoogwa vs. Ngoogwe | -7,193 | -36,74 to 22,36 | 0,9998 |
| Kagoogwa vs. Bire | 1,027 | -28,52 to 30,58 | >0,9999 |
| Kagoogwa vs. Doodo red | 2,927 | -26,62 to 32,48 | >0,9999 |
| MPI vs. Takataka | 43,06 | 13,51 to 72,61 | 0,0007 |
| MPI vs. Boribo | 40,71 | 11,16 to 70,26 | 0,0015 |
| MPI vs. Ngoogwe | 38,95 | 9,395 to 68,50 | 0,0026 |
| MPI vs. Bire | 47,17 | 17,62 to 76,72 | 0,0002 |
| MPI vs. Doodo red | 49,07 | 19,52 to 78,62 | <0,0001 |
| Takataka vs. Boribo | -2,347 | -31,90 to 27,20 | >0,9999 |
| Takataka vs. Ngoogwe | -4,110 | -33,66 to 25,44 | >0,9999 |
| Takataka vs. Bire | 4,110 | -25,44 to 33,66 | >0,9999 |
| Takataka vs. Doodo red | 6,010 | -23,54 to 35,56 | >0,9999 |
| Boribo vs. Ngoogwe | -1,763 | -31,31 to 27,79 | >0,9999 |
| Boribo vs. Bire | 6,457 | -23,09 to 36,01 | >0,9999 |
| Boribo vs. Doodo red | 8,357 | -21,19 to 37,91 | 0,9989 |
| Ngoogwe vs. Bire | 8,220 | -21,33 to 37,77 | 0,9990 |
| Ngoogwe vs. Doodo red | 10,12 | -19,43 to 39,67 | 0,9924 |
| Bire vs. Doodo red | 1,900 | -27,65 to 31,45 | >0,9999 |
